# Metabarcoding prey DNA from fecal samples of adult dragonflies shows no predicted sex differences, and substantial inter-individual variation, in diets

**DOI:** 10.7717/peerj.12634

**Published:** 2021-12-17

**Authors:** André Morrill, Kari M. Kaunisto, Julia J. Mlynarek, Ella Sippola, Eero J. Vesterinen, Mark R. Forbes

**Affiliations:** 1 Department of Biology, Carleton University, Ottawa, Ontario, Canada; 2 Biodiversity Unit, University of Turku, Turku, Finland; 3 Insectarium de Montreal, Montreal, Quebec, Canada; 4 Department of Ecology and Genetics, University of Oulu, Oulu, Finland; 5 Department of Biology, University of Turku, Turku, Finland; 6 Department of Ecology, Swedish University of Agricultural Sciences, Uppsala, Sweden

**Keywords:** Diet analysis, fDNA, Metabarcoding, Odonata, Prey species, Niche differentiation

## Abstract

Sexes often differ in foraging and diet, which is associated with sex differences in size, trophic morphology, use of habitats, and/or life history tactics. Herein, strikingly similar diets were found for adult sexes of a dragonfly (*Leucorrhinia intacta*), based on comparing 141 dietary taxa identified from the metabarcoding of mitochondrial DNA archived in feces. Arthropods in > 5% of samples included five species of dipterans, two hemipterans, two spider species and one parasitic mite. The mite was not traditional prey as its presence was likely due to DNA contamination of samples arising through parasitism or possibly via accidental consumption during grooming, and therefore the mite was excluded from diet characterizations. Common prey species were found with statistically indistinguishable frequencies in male and female diets, with one exception of an aphid more often found in male diets, although this pattern was not robust to corrections for multiple statistical tests. While rare prey species were often found in diets of only one sex, instances of this were more frequent in the more oft-sampled females, suggesting sampling artefact. Sexes did not differ in the mean prey species richness in their diets. Overall, sexes showed statistically indistinguishable diets both on a prey species-by-species basis and in terms of multivariate characterizations of diet composition, derived from presence-absence data of prey species analyzed via PERMANOVA and accumulation curves. Males and females may have similar diets by being both opportunistic and generalist predators of arthropods, using the same foraging habitats and having similar sizes and flight agilities. Notably, similarities in diet between sexes occur alongside large interindividual differences in diet, within sexes. Researchers intending on explaining adaptive sex differences in diet should consider characteristics of species whose sexes show similar diets.

## Introduction

By studying diet selection in animals, researchers can test ideas about trophic interactions and food web linkages (Nielsen et al., 2018; Kaunisto et al., 2020; Vesterinen, Kaunisto & Lilley, 2020) and about resource partitioning between or within species (Trevelline et al., 2018; Perkins, Cloyed & Eason, 2020; Li & Kokko, 2021). Resource partitioning within species might reduce competition between groups of individuals but might be more likely to occur as a consequence of differences in investments in growth, development and/or reproduction between immature and mature individuals or between males and females. Individual males and females, for example, could demand different food choices to fuel life history differences related to sexual size dimorphism (Shine et al., 2002) without the need to invoke reduced competition between the sexes as an explanation for any niche differentiation observed (see also Przybylo & Merilä, 2000). Sexes of particular insect species are known to differ substantially in their food choices because of sex differences in life histories. An obvious example is mosquitoes, where only the female is equipped to blood feed—vertebrate blood is necessary for the female to provision her eggs (Takken & Verhulst, 2013).

In predatory insects that feed on invertebrate prey, differences in diet composition between species or between conspecifics may be subtle and much more difficult to study. Direct observations of feeding by different species can uncover differences in types or quantity of prey consumed (*e.g.*, Corbet, 1999), as can diet analysis whereby less digestible body parts that have taxonomic value are removed from insect guts and identified (Pritchard, 1964). For vertebrates, tissue fatty acid analyses have been used to discern diets and niche overlap, *e.g.*, between the sexes (Beck, Iverson & Bowen, 2005). Although less specific than the other types of diet analyses, stable isotopes (*e.g.*, carbon, nitrogen, sulfur) can help discern diets and niche overlap of the sexes (*e.g.*, Robinson et al., 2010). Such techniques are also applied to invertebrates (*e.g.*, Grant, Robison & Fincke, 2014).

Recently, researchers have used genetic techniques like metabarcoding of mitochondrial DNA (mtDNA) obtained from feces to determine diet composition of insects (*e.g.*, Kaunisto et al., 2020) and other animals (*e.g.*, Ando et al., 2020).  Specific taxonomic markers like mitochondrial *cytochrome oxidase subunit I* (COI) can be amplified. The resulting sequence variants (*e.g.*, zero-radius operational taxonomic units (ZOTUs) or amplicon sequence variants (ASVs)) can be compared with public databases to determine taxa or species of prey consumed. There is some discussion as to whether read counts can be used to determine the relative frequency of particular prey consumed (*e.g.*, see Berry et al., 2017; Deagle et al., 2019; Lamb et al., 2019; Vesterinen, Kaunisto & Lilley, 2020), which is useful if diets are to be described quantitatively (Kaunisto et al., 2020).

In this study, we used metabarcoding of feces to identify ASVs of arthropod prey in diets of adult males and females of the dot-tailed whiteface dragonfly *Leucorrhinia intacta* (Hagen, 1861). Adult dragonflies are highly effective and generally opportunistic predators employing various foraging strategies: ‘fliers’ which hunt during nearly continuous flight, ‘perchers’ which make short sallying flights from perches to capture prey, and ‘gleaners’ which hover over and capture prey from plant surfaces (Corbet, 1999; Kaunisto et al., 2017). Despite these largely opportunistic foraging stategies, there were reasons to expect adult sexes of *L. intacta* to differ in their diets. First, males require enough energy for either territory maintenance or mate searching as transients—both mating tactics are expressed by individual males in this species (Waltz & Wolf, 1988). In comparison, females should require lipid and protein for egg provisioning (Jiménez-Cortés, Serrano-Meneses & Córdoba-Aguilar, 2012). Furthermore, foraging male dragonflies might be ‘time minimizers’ that must visit or return to territories or search for potential mates. This is presumably after shorter foraging time intervals than required by females to acquire ample resources to provision and mature large clutches of eggs (‘energy maximizers’; *cf.* (Anholt, Marden & Jenkins, 1991)). In fact, this species shows different pre-reproductive periods spent foraging with males becoming sexually mature in eight days whereas females become sexually mature in 12 days (Deacon, 1975, cited in Corbet, 1999). Although males and females weigh about the same at emergence (∼24 mg on average), females gain much more mass (54 ± 2.2 mg) than do males (37.0 ± 2.5 mg) by sexual maturity (Anholt, Marden & Jenkins, 1991).

There are also reasons to expect limited sex differences in diet. Males and females have the same trophic morphology, catch prey in the same way by gleaning or in flight, they show considerable size overlap, and can be caught foraging in the same habitat, similar to the sexes of other dragonflies (Corbet, 1999). The sexes of this species also do not seem to differ noticeably in their ability to evade netting (M.R. Forbes, 1988–2018, Pers. Obs.) which, though we do not currently have data to support this specific conjecture, might mean they have similar flight agility in pursuing and capturing prey. Even if time minimization by males *versus* energy maximization by females is occurring, these differences could select for sex differences in food quantity rather than differences in food types or quality.

We specifically used metabarcoding data to determine the diet composition for individual dragonflies, approximated by recording the frequency with which ASVs of various prey species (or taxa) are represented in feces. As described later, we scored presence-absence of prey species, based on reaching a threshold number of ASV reads for any arthropod taxa recovered. We then assessed overlap in prey species use between the sexes alongside inter-individual variation in diet. In addition to characterizing diets on a prey species-by-prey species basis (*i.e.,* comparing the frequencies of each prey taxa’s consumption between the sexes), we also considered the prey species richness of diets and the degree to which the adult sexes compare as generalist predators, feeding on the same assemblage of potential prey, by comparing diet richness accumulation curves. To assess inter-individual diet variation, we compared the average Bray–Curtis dissimilarities of diet composition for males and females. Our study thus considers multiple metrics for diet compositions to assess whether there is evidence of resource differentiation between the adult sexes of a dragonfly.

## Materials & Methods

### Sample collection and processing

We netted dragonflies in old fields and woods’ edges (in or near the East Field complex site under stewardship of the Queens University Biological Station), in Ontario, Canada within one kilometre of the coordinates 44°32′29″N, 76°22′17″W over five days in June (8th–12th), 2018. Feeding or roosting dragonflies were netted opportunistically most often individually with large butterfly nets to avoid injury (diameter of hoop > 0.3 m). Dragonflies were removed from nets and placed individually into numbered and clean glass vials. Glass vials were placed in a cool box while in the field. Dragonflies in vials were transported to the Queens University Biological Station where vials were briefly uncapped to let air into the vial. Dragonflies were housed for up to 24 h after which the vials were checked for feces and, if present, feces were processed as described below. Each dragonfly was frozen at −20 °C and later transported to Carleton University as part of a larger study (Kaunisto, Morrill & Forbes, 2018).

We were able to obtain fecal samples from 152 *L. intacta* individuals (103 females and 49 males; female *L. intacta* had a higher relative abundance at the study site; Kaunisto, Morrill & Forbes, 2018). Feces were collected from vials with sterilized tweezers. To untangle the diets of this focal species, we used established metabarcoding protocols for dragonflies and damselflies, building on earlier optimization (Aljanabi & Martinez, 1997; Kaunisto et al., 2017; Kaunisto et al., 2020). DNA extractions were carried out in Carleton University following our previous work (Kaunisto et al., 2017; Kaunisto et al., 2020). The DNA extracts were transported to the University of Turku, Finland. We also prepared a mock community containing DNA from six insects: *Allocotocera pulchella* and *Coenosia mollicula* (Diptera), *Grypotes puncticollis* (Auchenorrhyncha), *Phygadeuon* sp. (Hymenoptera), *Crambus heringiellus* and *Apamea remissa* (Lepidoptera). The mock community was included in the analysis and processed exactly the same way as any other sample (see Article S1 for details on the mock community preparation).

### Molecular analysis

We carried out a two-phase PCR protocol to construct the DNA libraries for high throughput sequencing (Vesterinen et al., 2016; Vesterinen et al., 2018). Moreover, we designed blocking primers for dragonflies to improve the recovery of the prey DNA compared to the predator DNA. Shortly, the locus-specific first-PCR primers were tailed with Illumina-specific linker-tags (tagF and tagR), which allowed the adapter attachment in the subsequent second PCR (library PCR). All the steps of the molecular work are described in detail below.

**Choosing the markers and blocking primer design:** We updated our earlier protocols regarding the primers. Firstly, the previous primer pairs (Zeale primers ZBJ-ArtF1c/ZBJ-ArtR2c (Zeale et al., 2011) and Ins16S-1F/Ins16S-1Rshort (Clarke et al., 2014)) have gained some criticism due to the (1) potential bias towards certain taxa (ZBJ primers criticized in *e.g.*, Clarke et al., 2014), and (2) the 16S region does not have a comprehensive or curated reference library available (Kaunisto et al., 2017). Thus, we applied two different non-overlapping primer pairs to amplify different regions of the mitochondrial COI gene to maximise the recovery of the prey taxa and to identify them with confidence. We amplified a 313 bp COI fragment with the primer pair mlCOIintF: 5′-GGW ACW GGW TGA ACW GTW TAY CCY CC-3′ (Leray et al., 2013) and jgHCO2198: 5′-TAI ACY TCI GGR TGI CCR AAR AAY CA-3′ (Geller et al., 2013). Another COI fragment of 180 bp was amplified with the primer pair LCO1-1490: 5′-GGT CAA CAA ATC ATA AAG ATA TTG G-3′ (Folmer et al., 1994) and CO1-CFMRa: 5′-GGW ACW RGW TGR ACW ITI TAY CCY CC-3′ (Jusino et al., 2019). The mlCOIintF/jgHCO2198 primer pair is referred hereafter as Leray and LCO1-1490/CO1-CFMRa as ANML. Of these primer pairs, the ANML amplifies almost entirely the same region as the Zeale primers used in a previous study (Zeale et al., 2011), to enable comparison across studies, but ensuring lower bias between prey taxa. The choice or primers was done based on the recent literature and previous work and experience of co-authors.

Both of the primer pairs in the current study likely amplify the predator in the current study (*L. intacta*); for this reason, we designed a blocking primer for both primer pairs as follows. We downloaded ten *L. intacta* COI sequences from BOLD systems (http://www.boldsystems.org) and examples of potential prey from widely different arthropod orders opportunistically from GenBank (http://www.ncbi.nlm.nih.gov/genbank/) where full mitochondria or at least full COI gene (including priming sites for LCO and HCO) were available. We aligned all sequences using Geneious MUSCLE plugin (Edgar, 2004; Kearse et al., 2012). Then, we annotated all primers into downloaded reference sequences and manually searched for optimal sites for blocking primers. Blocking primers (Leray primer blocker: Lint-Ler-Blk-F 5′-GTT TAT CCT CCA CTA GCT GGA GCC AT **[C3]**- 3′; ANML primer blocker: Leuint-B-R2, 3′-CCC AAA ACC TCC AAT TAT AAT AGG TAT AAC **[C3]**- 3′) were ordered from Macrogen Inc. (Seoul, South Korea) with a C3 modification in the 3′ end to prevent amplification in the PCR.

**First PCR (locus-specific amplification):** To increase the amplicon library diversity, there were four versions of each primer, so that they included so-called heterogeneity spacers between the linker-tag and the actual locus-specific oligo. The four versions for Leray were: 0 = no spacer, 1 = C, 2 = TC, or 3 = ATC. For ANML, the primer versions were: no spacer, T, AT, or CAT. First phase PCR was executed as two technical replicates per sample per primer pair, and mixed versions 0+2 for the first PCR replicate, and versions 1+3 for the second replicate. We used a reaction volume of 10 µl for all primer pair reactions. The reaction volume included 5 µl of 2 × MyTaq HS Red Mix (Bioline, UK), 3.6 µl of H_2_O, 100 nM of each primer (forward 0+2 or 1+3; and reverse 0+2 or 1+3), 4000 nM of the blocker (20x concentration compared to the primers; Leray: Lint-Ler-Blk-F; ANML: Leuint-B-R2), and 1 µl of DNA extract per each sample.

The PCR cycling protocols were:

Leray: 5 min at 95 °C, then 16 cycles at 95 °C for 10 s, 61 °C (decreased by 1 °C per cycle) for 60 s and 72 °C for 30 s, followed by 20 cycles at 46 °C for 60 s and 72 °C for 30 s, followed by 72 °C for 10 min.

ANML: 60 s at 95 °C, then 5 cycles at 95 °C for 60 s, 45 °C for 90 s and 72 °C for 90 s, followed by 35 cycles at 95 °C for 60 s, 50 °C for 90 s and 72 °C for 60 s, followed by 72 °C for 10 min.

**Second PCR (NGS library construction):** In the second PCR, we used a dual indexing approach where we tagged both forward and reverse primers with different indexing tags (Shokralla et al., 2015; Vesterinen et al., 2016; Vesterinen et al., 2018). Each sample included a unique index combination to identify the reads after sequencing. These index sequences differed at a minimum of five bases between indices. Library preparation followed Vesterinen et al. (2016) with small modifications: for a reaction volume of 10 µl, we mixed 5 µl of MyTaq HS RedMix, 500 nM of each primer (i7 and i5) and 3 µl of locus-specific PCR product from the first PCR phase. For PCR cycling, we used the following protocol: 4 min at 95 °C, then 15 cycles of 20 s at 95 °C, 15 s at 60 °C and 30 s at 72 °C, followed by 3 min at 72 °C.

After library construction, we pooled all indexed samples in equal volumes, separately for the two primer pairs (Leray and ANML) and separately for both replicates (1 and 2), and purified the sub-pools following Vesterinen et al. (2016) dual-SPRI-purification protocol. The sub-pool DNA profiles were confirmed by electrophoresis and BioAnalyzer 1600 (Agilent Technologies, Santa Clara, California, USA), and final concentrations were measured using Qubit Fluorometer (dsDNA HS Assay kit; Invitrogen). The Leray pool was sequenced together with a bacterial 16S pool in MiSeq v3 chemistry with 600 cycles and 2*300 bp paired-end read length. The ANML pool was sequenced separately in a MiSeq v2 chemistry with 300 cycles and 2*150 bp paired-end read length. Sequencing was performed on the Illumina MiSeq platform (Illumina Inc., San Diego, California, USA) by the Turku Centre for Biotechnology, Turku, Finland.

After sequencing, the reads separated by each original sample were uploaded on CSC servers (IT Center for Science, http://www.csc.fi) for bioinformatic analysis. First, primers were removed separately for R1 and R2 reads by using the Python program cutadapt so that the read order within each file was left intact to allow smooth merging in the subsequent steps (Martin, 2011). The subsequent bioinformatics followed the DADA2 pipeline, conducted in R (version 3.6.1; R Core Team, 2019), to define the ASVs separately for each primer set (Callahan et al., 2016), with some primer-specific enhancements and modifications based on trials with a small subset of data. For the filterAndTrim step, the maximum allowed expected errors (maxEE parameter) was set to 4, and the number of bases after which bases were truncated (trunclen parameter) was set to R1: 200, R2: 180 (Leray) and 115, 100 (ANML). The Leray set was represented by 3,064 ASVs in 3,297,660 reads, and the ANML set with 3,281 ASVs in 5,319,660 non-chimeric reads. ASVs were assigned to taxonomy with SINTAX algorithm as implemented in USEARCH using all the public sequences on BOLD Systems (http://www.boldsystems.org) with species identification (Edgar, 2010). Mock community sequences were included in the database. All the read counts for each sample were tracked throughout the pipeline (“input”, “filtered”, “merged”, “tabled”, “nonchim”; for the read counts in the DADA2 pipeline, see Table S1).

Next, the nonchimeric seqtabs (= ASV x sample matrices) were filtered for wrongly assigned reads based on the negative controls (extraction blanks and PCR negatives) by removing the reads assigned to an ASV from each sample where the read count was below the read count of any negative control. Then, we defined the reliable taxa identification thresholds based on the mock community data. Based on this, we used the following thresholds from the SINTAX probabilities: species < 0.45, genus < 0.4, family < 0.3, and order < 0.2. While these thresholds are rather low, we tested these with mock samples and for the data as a whole, and found no evidence of wrong assignments. We collapsed and summed up the reads of all ASVs that were identified to the same taxa within samples, and then removed prey taxa from the samples if only one of the replicates had produced reads. Then we removed all the matches to non-arthropods: in the Leray set ∼93% remained, and in the ANML ∼88% remained. The removed ASVs were analysed, and they consisted mainly of microscopic Fungi and some Bacteria, such as Rickettsiales, known endosymbionts of arthropods. To estimate the proportion of reads that could have been misassigned during index demultiplexing (known as ‘tag-jumping’ or ‘sample cross-talk’), we calculated the proportion of non-mock reads (ANML: 4,010 reads; Leray: 1,866) out of the total number of reads per mock samples (ANML: 493,326 reads; Leray: 359,600). The estimation of tag-jumping was made independently for each primer pair dataset. This revealed a tag-jumping rate per primer pair (0.82% for ANML; 0.52% for Leray). Then, we removed any ASV with a proportion of reads less than the specified tag-jump rate of the total read sum of the sample-specific read number. The blocking primers seemed to work perfectly for both data sets. After all filtering, the Leray set had altogether 2,544,092 reads, of which 97.2% were prey reads and the rest were from the predator or unidentified. The ANML set consisted of 3,859,179 reads, of which 99.8% were prey reads, and the rest unidentified. We pre-analysed that both datasets gave similar results, and then combined both datasets together. All the mock species assignations were removed prior the subsequent analysis. The final dataset (3,859,179 reads) was turned into presence/absence data of particular prey species; the relative read abundance data were not used to quantify the diet further, *i.e.,* by ascribing a particular frequency of biomass to particular prey items (cf. Kaunisto et al., 2020). Thus, in our datasets, we only consider that the presence of a barcode means a specific prey item was present in the diet sometime in the past, and we did not gauge at what relative abundance that prey was consumed for an individual dragonfly. We did evaluate the prevalence of prey in the diet, *i.e.,* whether a particular prey item was frequently ascribed as being present or not across individuals, and we could then compare the relative frequency of individuals in a particular category (say, males) known to have fed on that prey item. Feces from 99 females and 48 males provided threshold reads for at least one arthropod taxon.

### Statistical analyses

Following quantification and filtering of ASV reads, all analyses were performed in R (version 3.6.1; R Core Team, 2019).

Possible differences in diet between male and female dragonflies were analyzed first by comparing the proportional representation of individual prey types (ASVs), then of prey aggregated by higher taxonomic level (*e.g.*, insect Order), and finally by comparing overall accumulation curves, prey community compositions, and average prey species richness. Each approach is described in turn.

The proportions of each of the ten most prevalent prey types (ASVs; ranging from 5.9% to 40.8% of diets) were compared between the sexes using Fisher’s exact tests. Evidence for differences between males and females in prevalences of prey type Orders was assessed informally by checking whether 95% Clopper–Pearson confidence intervals, of prevalence estimates for that prey type, overlapped.

We generated diet richness accumulation curves for each sex by measuring cumulative diet richness (from ASVs present) across considered samples after each of 1,000 randomizations of those samples. These allowed us to evaluate whether our methods likely captured close to the total richness of the diet for sexes of this predatory dragonfly (*i.e.,* whether the curves approached or had met an apparent horizontal asymptote). We also used accumulation curves to test whether the diets of males and females were likely drawn from a single (identical) ‘assemblage’ of possible prey. To do this, we compared the areas between group- (in this case, sex-) specific curves and a combined total accumulation curve to a null distribution of analogous differences in area when randomly assigning individuals to groups in repeated reshufflings (maintaining the sizes of the underlying groups; Cayuela, Gotelli & Colwell, 2015). This was accomplished using the EcoTest.sample() function from the ‘rareNMtests’ package, using 200 iterations (Cayuela & Gotelli, 2014). We attempted to extrapolate population-level estimates of total diet richness using the poolaccum() function from the ‘vegan’ R package, though these results are not reported as the algorithms showed no signs of having properly converged (Oksanen et al., 2019).

Overall diet composition was also compared between males and females by considering the average Bray Curtis dissimilarities between the groups of samples, calculated from presence-absences ((A + B −2  × J)/(A + B), where A and B are the number of prey in each compared diet, and J is the number of prey present in both diets; (Oksanen et al., 2019)). Differences in diet compositions were analyzed using permutational multivariate analysis of variance (PERMANOVA, 10,000 permutations; adonis() function from the ‘vegan’ package; Oksanen et al., 2019). To confirm that any significant results from the PERMANOVAs were reflecting a difference in diet composition that did not arise simply from differences in multivariate spread of dissimilarities between the sexes, we tested this assumption using an analysis of multivariate homogeneity of group dispersions (betadisper() function in the ‘vegan’ package; Oksanen et al., 2019). Finally, we compared basic diet richness between males and females using Mann–Whitney-Wilcoxon tests. Reported 95% confidence intervals around average diet richness and dissimilarity measures are bias-corrected and accelerated (BCa) bootstrap intervals. In all tests, statistical significance was assessed at α = 0.05. When multiple similar hypotheses were tested (*i.e.,* the Fisher’s exact tests), we applied the Holm-Bonferroni method to reduce the probability of false positives.

We considered the parasitic mite *Arrenurus reflexus* in the first analysis of common invertebrates barcoded from feces but excluded *A. reflexus* from all diet characterizations. This was done for two main reasons. First, the mite is a parasite known to attach to the abdomen of males and females (males were more frequently parasitized in our sample; Kaunisto, Morrill & Forbes, 2018); it is possible that mite DNA contamination of fecal material can account for the significant sex bias in representation of this arthropod in male feces (see Results), as parasitic water mites generate a specialized feeding tube (a stylostome) through the host’s exoskeleton. This is the most parsimonious explanation. Second, the mite DNA might have resulted from dragonflies grooming off engorging mites and consuming some or all of a mite(s) on occasion, but this is not really a ‘prey’ item in a conventional sense. Regardless of which of the two explanations is more likely, any resulting sex bias would not reflect differences in prey preferences or feeding behaviours between the sexes, but would be simply a consequence of an initial sex bias in infection levels. We briefly consider in the Discussion how it is possible that mites were ingested along with conspecifics (*cf*. Nagel, Zanuttig & Forbes, 2011) due to cannibalism with cannibalistic activities being higher for males than females; the blocking primers would not pick up such cannibalistic activity.

## Results

Adult males and females of the dragonfly *L. intacta* are both generalist predators feeding on a wide array of insect and other arthropod prey. In total, 141 uniquely barcoded species or ASVs were identified as ‘prey’ from the feces of *L. intacta* dragonflies (Table 1; Fig. S1). Those species ranged from being found in only one of 147 samples (on 79 separate occasions) to a spider species *Schizocosa saltatrix* being found in 40.8% (95% CI [32.9%–49.0%]) of fecal samples that were analysed (Table 1; Fig. S1).

**Table 1:** Ten overall highest-prevalence individual taxa detected in the diets of *Leucorrhinia intacta* dragonflies. Overall prevalence and within-sex prevalence is provided. Clopper–Pearson 95% confidence intervals are in parentheses.

Prey taxa	Order	Overall	Female	Male
*Schizocosa saltatrix*	Araneae	0.408 (0.329–0.49)	0.417 (0.321–0.519)	0.388 (0.252–0.538)
*Arrenurus reflexus* ^a^	Trombidiformes	0.211 (0.149–0.284)	0.155 (0.091–0.24)	0.327 (0.199–0.475)
*Peyerimhoffia vagabunda*	Diptera	0.132 (0.082–0.196)	0.155 (0.091–0.24)	0.082 (0.023–0.196)
*Macrosteles patruelis*	Hemiptera	0.125 (0.077–0.188)	0.146 (0.084–0.229)	0.082 (0.023–0.196)
*Ablabesmyia illinoensis*	Diptera	0.112 (0.067–0.173)	0.126 (0.069–0.206)	0.082 (0.023–0.196)
*Platypalpus sp.*	Diptera	0.092 (0.051–0.15)	0.097 (0.048–0.171)	0.082 (0.023–0.196)
*Minettia lyraformins*	Diptera	0.086 (0.046–0.142)	0.087 (0.041–0.159)	0.082 (0.023–0.196)
*Eriosoma americanum*	Hemiptera	0.072 (0.037–0.126)	0.039 (0.011–0.096)	0.143 (0.059–0.272)
*Schizocosa sp.*	Araneae	0.066 (0.032–0.118)	0.049 (0.016–0.11)	0.102 (0.034–0.222)
*Smittia sp.*	Diptera	0.059 (0.027–0.109)	0.049 (0.016–0.11)	0.082 (0.023–0.196)

**Notes.**

a Possibly due to contamination of fecal samples by detaching parasitic mites and not a diet component at all unless sufficient mite tissue body parts ingested by grooming. That this taxon is more likely reported from males accords well with the sex biases in parasitism shown for samples of this species.

The ten most common ASVs retrieved from metabarcoding feces included the aforementioned spider (*S. saltatrix*), a parasitic mite (*Arrenurus reflexus*) found in 21.1% of samples (disregarded during subsequent analyses; see above), five Dipteran species found in 13.2, 11.2, 9.2, 8.6, and 5.9% of samples, two Hemipterans found in 12.5 and 7.2% of samples and another *Schizocosa* spider found in 6.6% of samples (Table 1; see this table also for confidence limits around prevalence estimates, named species and genera, and prevalence estimates of prey items partitioned by sex). Fisher’s exact tests identified two common species that differed in proportional representation between the sexes at *α* = 0.05: both the parasitic mite *A. reflexus* (*p* = 0.02) and the wooly elm aphid *Eriosoma americanum* (*p* = 0.039) demonstrated higher representation in male as compared to female fecal samples. However, neither of these differences remained significant after controlling for the multiple (ten) comparisons using the Holm-Bonferroni method. Comparing the remaining 131 ASVs between the dragonfly sexes told a similar story to that of the majority of common prey. If a prey item was present in samples of females, it was also nearly equally present in samples of males, unless the prey item was rare overall in which case it could be found in just one or two samples of either sex, but this more commonly occurred in samples of the more often sampled females (Fig. S1).

**Figure 1:**
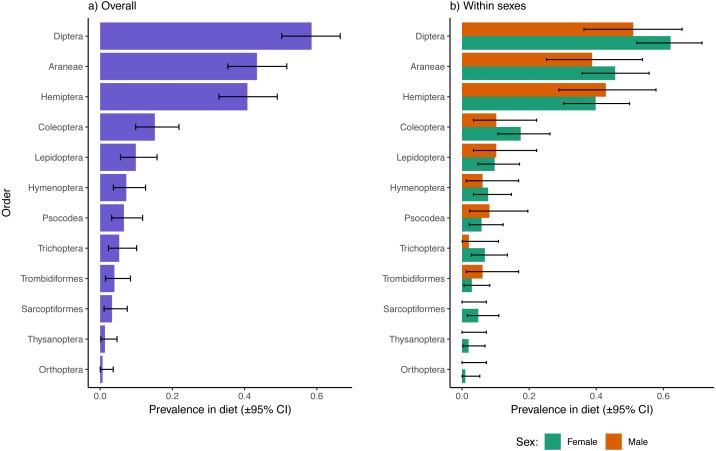
Prevalence of different insect and invertebrate orders in the diets of *Leucorrhinia intacta* dragonflies overall (A) and partitioned by sex (B). Clopper–Pearson 95% confidence intervals are provided. Reads of *Arrenurus reflexus*, a parasitic mite, were disregarded as these likely represented DNA contaminations from infecting mites, or arose from consumption of mites removed during grooming (either way, not representative of differences in feeding preferences between the sexes).

Excluding reads of the parasitic mite, Dipterans were the most well represented prey type in fecal samples overall (58.6% of samples had one or more Dipteran species represented), followed by Araneae (43.4%), Hemiptera (40.8%), and Coleoptera (15.1%; Fig. 1A). The 95% confidence intervals of Diptera, Araneae, and Hemiptera did not overlap with any of the remaining observed orders, indicating that other orders were significantly less represented in dragonfly diets. There were no apparent sex biases in representation of prey from these higher taxonomic levels in the diets of dragonflies (Fig. 1B). There were also no sex differences in dietary inclusion of representatives from Lepidoptera, Hymenoptera, Psocodea, Trichopera, Trombidiformes (*A. reflexus* removed), Sarcoptiformes, Thysanoptera, or Orthoptera (Fig. 1B), all of which were each found in < 10% of samples overall (Fig. 1A).

We also compared the average number of uniquely coded prey (akin to species richness) found in samples of male and female diets. On average, female diets contained 3.18 (95% CI [2.73–3.79]) prey species whereas male diets had 3.02 (2.38–3.76) species, a result that was not statistically significant (Mann–Whitney-Wilcoxon *Z* = 0.22, *p* = 0.82).

**Figure 2:**
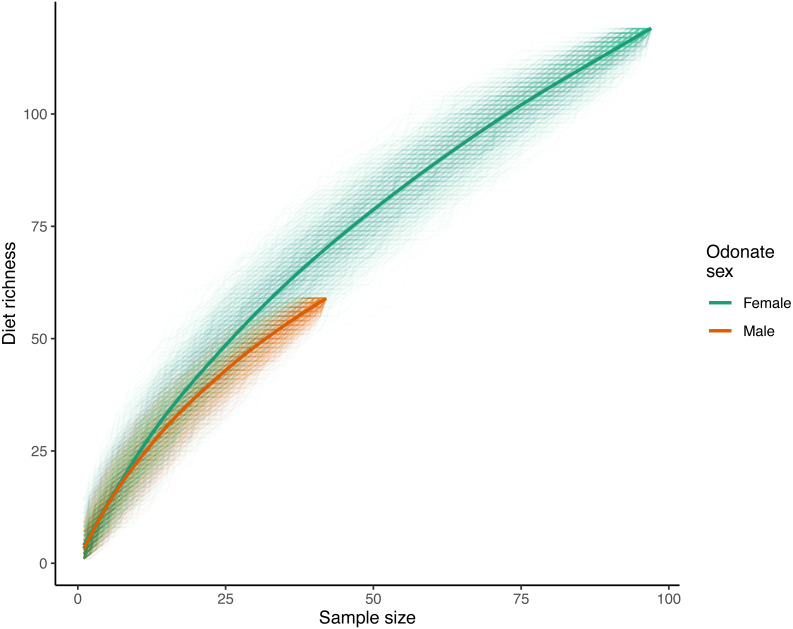
Diet richness accumulation curves for male and female samples of *Leucorrhinia intacta*. Cumulative diet richness for 1,000 random orderings of the samples within each sex are shown with translucent lines. The solid smoothed lines were fit using a generalized additive model with a cubic regression spline on the covariate sample size (R function mcv::gam()), where the response was each of the individual cumulative richnesses across all the randomizations.

With respect to species accumulation curves, a total of 119 unique prey ASVs were identified in female samples while 59 were recorded in males (140 ASVs total with the mite *A. reflexus* removed). Note that the sample size for females was much higher than that of males (97 and 42 respectively after removing samples where the only recorded reads were from *A. reflexus*; Fig. 2). Diet richness accumulation curves for each sex showed no signs of approaching asymptotes (Fig. 2); therefore, the actual total richness of *L. intacta* diets in this population is likely higher than reported herein. Extrapolated estimates of total diet richness were not identified as estimation algorithms showed no evidence of properly converging.

Analysis of diet accumulation curves showed no evidence that diets of male and female *L. intacta* came from different potential prey ‘assemblages’ rather than from one single pool (randomization test *p* = 0.57).

Overall average Bray–Curtis dissimilarities between individual diets based on prey presence-absences was 0.92 (95% CI [0.91–0.92]). Analyses of Bray–Curtis dissimilarities showed no evidence of differences between the sexes, neither in terms of multivariate spread (*F*_1,137_ = 0.05, *p* = 0.83) nor underlying prey composition (PERMANOVA *F*_1,137_ = 1.07, *p* = 0.35, *R*^2^ = 0.01).

## Discussion

Our results show that both sexes of the dragonfly *L. intacta* are generalist predators of arthropods and show extreme overlap in inclusion of particular common prey items in their diets. Additionally, diet compositions based on higher taxonomic levels (*e.g.*, insect Order) did not differ appreciably between males and females and ‘sex differences’ in diets based on rare ASVs found in the diets of only one of the sexes could be explained by chance occurrences. Sexes also did not differ in average prey species richness of their diets. We discovered, using prey richness accumulation curves, that prey included in diets were only a subset of prey from the same arthropod assemblage expected to be preyed upon by males and females of this species. We further found that there is considerable interindividual variation in diets. We discuss the implications of our study in light of research attempting to uncover adaptive niche differentiation between the sexes, while also entertaining specific technical and other issues and commenting on the natural history of this species vis-à-vis its foraging ecology.

The first point is that males and females did not differ in dietary inclusion of specific prey items that were relatively common. These dietary indifferences between the sexes were based on prey presence-absence data rather than abundance data and followed conservative statistical corrections for multiple tests. It is possible that sex differences in abundance of particular common prey items would have been revealed had abundance of particular prey taxa been indexed. Using relative read numbers as an index for prey numbers requires translating aggregate ASV read numbers into an aggregate biomass for a particular prey taxon and dividing those values by average biomass for individuals of that prey taxon to estimate prey numbers (*e.g.*, Deagle et al., 2019). Indexing abundance of prey items using ASV read number also assumes that relative rates of amplification of COI barcodes is similar for different taxa or has been assessed for different taxa directly through experimentation (Clarke et al., 2014). Such ‘corrections’ were beyond the scope of the present study. As mentioned previously, we took a conservative approach and considered a prey taxon to be present if its ASV exceeded a threshold number of reads (see Methods). Even if tests based on presence-absence data are less powerful, we still have multiple common and many rare species to be assessed for dietary differences between the sexes.

If we ignore statistical corrections for multiple tests for the moment, we have two ‘prey’ taxa that were returned more often in male than in female fecal samples: the parasitic mite and the wooly elm aphid. For reasons of parsimony discussed already, the parasitic mite was not considered prey. Some of its occurrence could have resulted from dragonflies feeding on parasitized conspecifics cannibalistically. *Arrenurus reflexus* is a common parasite of *L. intacta* (Mitchell, 1967). This occurrence of secondary predation would not be detected herein because of the blocking primer used for *L.intacta* mtDNA to prevent detection of sampled predator reads. Although cannibalism is known to occur in odonates (Corbet, 1999), cannibalism among adult *L. intacta* and its possible expression more frequently by males cannot be inferred from our data. Other simpler explanations exist for the greater occurrence of *A. reflexus* reads among male samples. That males were more parasitized than females in our sample (Kaunisto, Morrill & Forbes, 2018) may be because samples were comprised of younger males whose mites had not yet detached and older females whose mites had detached before capture (Mitchell, 1969). This male bias in current parasitism might explain the male bias in mite reads, if mites were ingested by grooming or mites detached in vials and mite mtDNA contaminated fecal samples, as mentioned already.

The marginally significant male bias in wooly elm aphid ingestion is more difficult to explain. Notably, wooly aphids also were part of the female’s diet, albeit less so (Table 1). Aphids are likely gleaned from plants by dragonflies, although aphids could also be captured in flight. Differences in *L. intacta* flight agility of the sexes, even if they exist, would be unlikely to result in different capture efficiencies of these slow-flying insects. For comparison, the sexes did not differ in ingestion of wolf spiders which are expected to be caught as slow-‘flying’ ballooning spiderlings, although this possibility needs to be investigated more fully. The sexes are likely opportunistic with respect to also feeding on wooly elm aphids and showed only chance differences in representation of this prey item in their diets.

As mentioned, there were no sex differences in inclusion of rare prey items that could not otherwise be easily explained by chance occurrences (*i.e.,* we did not find more instances of rare prey items in diets of the less frequently sampled males). The lack of sex differences in ingestion of prey at higher taxonomic levels (*e.g.*, insect Order) was expected, given no consistent sex differences in frequency of ingestion of the many other particular prey taxa (Fig. S1, Table 1). It seems that males and females equally feed on dipterans of a particular size range and availability as the top four dipterans species included in diets all range in size from 2 to 5 mm.

That there were no differences in prey species richness of male and female diets is interesting. This metric might be expected to differ between the sexes if the sexes differ in time spent foraging and/or in gut fullness upon capture; additional prey taxa would be expected in the diets of individuals having fed more. Species richness in diets is perhaps too conservative a metric to be useful in drawing inferences of foraging differences between the sexes; other measures of diversity accounting for different abundances of prey rather than simple presences/absences may be more informative. Taken collectively, tests were conservative, but many prey species were considered and there was limited to no evidence of diet differentiation between the sexes, considered as groups of individuals.

The evaluations of species or taxa accumulation curves for the sexes provide an additional insight; that is, the total prey species richness ingested by these dragonflies is likely much greater than reported herein (see Gotelli & Chao, 2013). One would need a much larger sample size and multiple sampling sites to adequately capture the full diversity of consumed prey for either sex. This difficulty in capturing the full richness of a prey assemblage even when given a seemingly large sample size may not be uncommon in dietary studies of generalist predators using metabarcoding approaches (see Corse et al., 2017; Sullins et al., 2018; Van Zinnicq Bergmann et al. 2020), and researchers should keep this in mind when designing their studies and forming their inferences. Given the similarity of the sex-specific curves observed herein despite obvious differences in sample sizes, it seems that male and female *L. intacta* are consuming prey from the same underlying assemblage of species or taxa. This finding further supports the contention of dietary overlap between groups of males and females, even though not all potential prey taxa have been accounted for. Interestingly, no blood-sucking mosquitoes were found in the diets of any sampled dragonflies, despite odonates having been found to consume mosquito pest species in other studies (*e.g.*, Córdoba-Aguilar et al. 2021). Mosquitoes may well be included in the unobserved portion of the *L. intacta* prey, as blood-sucking mosquitoes are certainly present at the study site (AM, KMK, JJM, MRF, Pers. Obs.).

It is important to reiterate that sampling occurred over five days for a species that has a flight season of over three months in Eastern Ontario, Canada (Paulson, 2011; MRF, Pers. Obs.). We fully expect seasonal variation in dietary composition in this species, and we cannot rule out that sex differences in diet composition may emerge when a different assemblage of potential prey is available (*c.f.* Córdoba-Aguilar et al., 2021). Additionally, while sampling of teneral individuals was avoided, there was no strict control guaranteeing that sampled individuals were of a specific age; it may be that underlying age distributions differ between the sexes given our selected habitat (fields and woods’ edges; Kaunisto, Morrill & Forbes, 2018) in ways that would affect diet composition, although this did not emerge as any observable sex differences in diet in this study.

Finally, we addressed inter-individual variation in diets, based on Bray–Curtis dissimilarities. A useful adage to remember is that tests of differences between groups are made while considering variation within groups. Males and females of this species show similar variation in diet among individuals (within groups) and no detectable difference between the sexes (groups) in the average diet dissimilarity. Taken collectively, our results suggest strongly that sexes (groups) of this dragonfly feed on the same prey species with equal frequency, but that individual diets can vary substantially.

Our results are perhaps not surprising. After all, males and females of this species overlap in size, have the same trophic morphology, seem to have the same flight agility and can be caught foraging in the same habitats and only differ in time minimization *versus* energy maximization tactics (see Introduction). We can add to this retinue that males and females are generalist, and very likely opportunistic, predators, which are presented with a diverse assemblage of potential arthropod prey, some of which are predators themselves, opening the possibility of secondary predation and increased ‘prey’ diversity. Additionally, differences between the sexes’ foraging tactics may have lessened post-reproductive age, for example, if the older females sampled in this study had relatively short inter-clutch intervals or matured smaller clutches of eggs (in other words, a hypothetical relative lessening of the energy maximization strategy). Recently, researchers have questioned which factors might drive sexual niche differentiation including limited genetic constraint, mating system, and spatial variation in resources (Li & Kokko, 2021). Sexual niche differentiation with respect to prey is also absent from other odonate species (Kaunisto et al., 2017; Kaunisto et al., 2020). With a diverse prey base, perhaps there are costs to specialization on particular prey items by individuals regardless if these are males or females. This generalist strategy might account for both inter-individual variation in diet and sharing of prey species between male and female diets. Whether this observation of a single prey assemblage shared between sexes is robust to considerations of abundance of consumed prey, and whether it then extends to other odonate species, could inform sampling procedures in studies of dragonfly and damselfly diets. For example, if there were no underlying differences in diets, collections could focus on males where females were either less abundant or more difficult to locate.

## Conclusions

Known sex differences in *L. intacta* pre-reproductive periods, in nutritional/energetic requirements, and in reproductive behaviours were expected to translate into sex differences in diet. However, the composition and diversity of *L. intacta* prey were not significantly different between adult males and females, while inter-individual variation in diet was high. Researchers testing for sex differences in diets should consider that highly opportunistic generalist predators may not demonstrate distinct patterns of prey consumption, even given underlying differences in life history traits.

There are still life history differences between the sexes that could translate into differences in diets that were not detectable, and as mentioned, there is the possibility that sexes differ in diet quantity. This possibility could not be adequately addressed using our data. Future research should compare gut fullness measures between males and females that are first returning to mating sites to defend territories (males) or lay eggs (females). Second, the sexes might differ in their assimilation of protein and lipids from their diet and this might be reflected in sex differences in the microbiome. Any differences in core microbiota might thus reflect differences in investment in immunity or assimilation. This intriguing idea will have to await formal study of microbiota similarities or differences between the adult sexes.

## Supplemental Information

10.7717/peerj.12634/supp-1 Supplemental Information 1 Read counts per sample through the DADA2 pipelin The **input** column refers to the input read numbers after trimming the primers. **Filtered** stands for reads after filtering. **Merged** reports how many of the reads were merged. **Tabled** means how many of the filtered reads were mapped to the seqtab (sequence table, similar to otutable) and the **nonchim** is the final number of reads in the denoised, non-chimeric seqtab. **PropRetained** is the proportion of the input reads which was retained in the nonchimeric seqtab.

10.7717/peerj.12634/supp-2 Supplemental Information 2 Details on mock community preparation

10.7717/peerj.12634/supp-3 Supplemental Information 3 Prevalences of individual prey identified as amplicon sequence variants within the diets of sampled male and female *Leucorrhinia intacta*, grouped by taxonomic order This excludes the ten most prevalent amplicon sequence variants; see main text and Table 1. Clopper–Pearson 95% confidence intervals are provided.

## Additional Information and Declarations

### Data Availability

The following information was supplied regarding data availability: The data is available at the Scholars Portal Dataverse: Morrill, Andre, 2021, “Adult dragonfly (*Leucorrhinia intacta*) sample data, and both taxonomic classifications and read counts from metabarcoding of dietary taxa using fDNA”, https://doi.org/10.5683/SP2/B8YGKG, Scholars Portal Dataverse, V1, UNF:6:HIMzaHiLQBkUA9Svp2lvHw== [fileUNF]
