# Supplementary material for: Metabarcoding prey DNA from fecal samples of adult dragonflies shows no predicted sex differences, and substantial inter-individual variation, in diets
Source: PeerJ. 2021 Dec 17;9:e12634. doi: 10.7717/peerj.12634 (PMC8686731; doi:10.7717/peerj.12634)
Supplement: Supplemental Information 1 — The input column refers to the input read numbers after trimming the primers. Filtered stands for reads after filtering. Merged reports how many of the reads were merged. Tabled means how many of the filtered reads were mapped to the seqtab (sequence table, similar to otutable) and the nonchim is the final number of reads in the denoised, non-chimeric seqtab. PropRetained is the proportion of the input reads which was retained in the nonchimeric seqtab. [file peerj-09-12634-s001.pdf]

Supplementary Table S1: Read counts per sample through the DADA2 pipeline

Supplementary Table S1 for

Metabarcoding prey DNA from fecal samples of adult dragonflies shows no predicted sex differences, and substantial inter-individual variation, in diets

André Morrill, Kari M. Kaunisto, Julia J Mlynarek, Ella Sippola, Eero J Vesterinen, and Mark R. Forbes

Corresponding author: André Morrill (andre_morrill@carleton.ca)

In the DADA2 pipeline, the read counts for each sample are tracked throughout the analysis. Here, we provide the numbers per each primer pair.

Table S1-1: Reads for ANML primer set. The **input** column refers to the input read numbers after trimming the primers. **Filtered** stands for reads after filtering. **Merged** reports how many of the reads were merged. **Tabled** means how many of the filtered reads were mapped to the seqtab (sequence table, similar to otutable) and the **nonchim** is the final number of reads in the denoised, non-chimeric seqtab. **PropRetained** is the proportion of the input reads which was retained in the nonchimeric seqtab.

Sample	input	filtered	merged	tabled	nonchim	PropRetained
**ANML-1-Leu-108**	50375	50363	28609	28609	26184	0.51978164
**ANML-1-Leu-118**	24821	24818	17954	17954	17656	0.71133315
**ANML-1-Leu-128**	13281	13275	3667	3667	3667	0.27610873
**ANML-1-Leu-138**	25998	25995	10559	10559	10049	0.38652973
**ANML-1-Leu-148**	3324	3323	461	461	461	0.13868833
**ANML-1-Leu-158**	40418	40406	31487	31487	29865	0.73890346
**ANML-1-Leu-168**	23763	23758	11481	11481	10624	0.4470816
**ANML-1-Leu-178**	23554	23543	5366	5366	5306	0.22526959
**ANML-1-Leu-18**	38860	38852	22828	22828	22068	0.56788471
**ANML-1-Leu-188**	46924	46915	34370	34370	32189	0.68598159
**ANML-1-Leu-198**	8314	8312	331	331	331	0.03981236
**ANML-1-Leu-208**	22531	22526	15015	15015	14713	0.65301141
**ANML-1-Leu-218**	36903	36894	19090	19090	16601	0.44985503
**ANML-1-Leu-228**	11408	11402	886	886	886	0.0776648
**ANML-1-Leu-238**	13298	13297	2614	2614	2614	0.19657091
**ANML-1-Leu-248**	7974	7973	30	30	30	0.00376223
**ANML-1-Leu-258**	23805	23802	9094	9094	9089	0.38181054
**ANML-1-Leu-268**	40563	40556	16940	16940	16706	0.41185317
**ANML-1-Leu-278**	11288	11284	2535	2535	2535	0.22457477
**ANML-1-Leu-28**	14508	14508	2703	2703	2703	0.186311
**ANML-1-Leu-288**	14198	14196	3237	3237	3237	0.22798986
**ANML-1-Leu-38**	9690	9689	412	412	412	0.04251806

Supplementary Table S1: Read counts per sample through the DADA2 pipeline

Sample	input	filtered	merged	tabled	nonchim	PropRetained
**ANML-1-Leu-48**	11711	11710	3545	3545	3545	0.30270686
**ANML-1-Leu-8**	28877	28800	24040	24040	24014	0.83159608
**ANML-1-Leu-98**	42233	42226	22293	22293	20835	0.4933346
**ANML-2-Leu-108**	445	445	271	271	271	0.60898876
**ANML-2-Leu-118**	124	124	17	17	17	0.13709677
**ANML-2-Leu-128**	105	105	0	0	0	0
**ANML-2-Leu-138**	251	251	110	110	110	0.43824701
**ANML-2-Leu-148**	42114	42110	21923	21923	17724	0.42085767
**ANML-2-Leu-158**	23260	23228	18173	18173	17513	0.75292347
**ANML-2-Leu-168**	19781	19766	2903	2903	2863	0.14473485
**ANML-2-Leu-178**	20556	20553	2594	2594	2594	0.12619187
**ANML-2-Leu-18**	175	175	41	41	41	0.23428571
**ANML-2-Leu-188**	19957	19939	3179	3179	3163	0.15849076
**ANML-2-Leu-198**	11796	11793	412	412	412	0.03492709
**ANML-2-Leu-208**	30061	30059	14438	14438	14152	0.47077609
**ANML-2-Leu-218**	44349	44345	20770	20770	18545	0.4181605
**ANML-2-Leu-228**	15286	15282	1880	1880	1880	0.12298836
**ANML-2-Leu-238**	14894	14886	2429	2429	2429	0.16308581
**ANML-2-Leu-248**	13816	13816	39	39	39	0.00282281
**ANML-2-Leu-258**	43901	43891	19859	19859	19382	0.44149336
**ANML-2-Leu-268**	28185	28176	7443	7443	7443	0.26407664
**ANML-2-Leu-278**	17635	17631	3730	3730	3730	0.2115112
**ANML-2-Leu-28**	191	191	25	25	25	0.13089005
**ANML-2-Leu-288**	13187	13184	284	284	284	0.02153636
**ANML-2-Leu-38**	119	119	0	0	0	0
**ANML-2-Leu-48**	116	116	19	19	19	0.1637931
**ANML-2-Leu-8**	71	71	19	19	19	0.26760563
**ANML-2-Leu-98**	194	194	43	43	43	0.22164948

Supplementary Table S1: Read counts per sample through the DADA2 pipeline

Table S1-2: Reads for Leray primer set. The **input** column refers to the input read numbers after trimming the primers. **Filtered** stands for reads after filtering. **Merged** reports how many of the reads were merged. **Tabled** means how many of the filtered reads were mapped to the seqtab (sequence table, similar to otutable) and the **nonchim** is the final number of reads in the denoised, non-chimeric seqtab. **PropRetained** is the proportion of the input reads which was retained in the nonchimeric seqtab.

Supplementary Table S1: Read counts per sample through the DADA2 pipeline

Sample	input	filtered	Merged	tabled	nonchim	PropRetained
Leray-1-FWM-10	5767	5275	5253	5253	5253	0.9108722
Leray-1-FWM-2	39742	38112	37168	37168	36683	0.92302853
Leray-1-FWM-3	48118	45098	37170	37170	31945	0.66388877
Leray-1-FWM-6	31695	26894	26723	26723	26723	0.84312983
Leray-1-FWM-7	38171	36581	36440	36440	36440	0.95465144
Leray-1-FWM-9	29030	27260	26985	26985	26985	0.92955563
Leray-1-Leu-1	3614	44	18	18	18	0.00498063
Leray-1-Leu-10	5885	211	191	191	191	0.0324554
Leray-1-Leu-100	12620	2923	2720	2720	2538	0.20110935
Leray-1-Leu-101	42995	17401	16820	16820	15032	0.34962205
Leray-1-Leu-102	48080	25867	24287	24287	21425	0.44561148
Leray-1-Leu-103	29649	12737	12254	12254	11679	0.39390873
Leray-1-Leu-104	7525	6329	6238	6238	6238	0.8289701
Leray-1-Leu-105	35755	23888	23410	23410	20367	0.56962663
Leray-1-Leu-106	10906	2278	2177	2177	2177	0.19961489
Leray-1-Leu-107	28068	10630	10496	10496	10467	0.37291578
Leray-1-Leu-108	51109	40254	38666	38666	32166	0.62936078
Leray-1-Leu-109	6357	78	7	7	7	0.00110115
Leray-1-Leu-11	38833	26053	25492	25492	23497	0.60507816
Leray-1-Leu-110	43051	19073	18482	18482	16394	0.38080416
Leray-1-Leu-111	49188	39079	38792	38792	38674	0.78624868
Leray-1-Leu-112	26990	10449	10007	10007	8929	0.33082623
Leray-1-Leu-113	17677	14766	14704	14704	14704	0.83181535
Leray-1-Leu-114	28258	9300	8965	8965	8880	0.31424729
Leray-1-Leu-115	15631	3985	3925	3925	3925	0.25110358
Leray-1-Leu-116	9830	1074	989	989	989	0.10061038
Leray-1-Leu-117	4616	261	241	241	241	0.05220971
Leray-1-Leu-118	4293	278	228	228	228	0.05310971
Leray-1-Leu-119	46064	39140	37965	37965	34006	0.73823376
Leray-1-Leu-12	7189	49	15	15	15	0.00208652
Leray-1-Leu-120	4616	403	369	369	369	0.07993934
Leray-1-Leu-121	20164	5223	4806	4806	3776	0.18726443
Leray-1-Leu-122	7416	160	133	133	133	0.0179342
Leray-1-Leu-123	5908	104	86	86	86	0.01455653
Leray-1-Leu-124	23913	7366	7036	7036	7013	0.29327144
Leray-1-Leu-125	7747	973	922	922	922	0.11901381
Leray-1-Leu-126	8127	1688	1482	1482	1482	0.18235511
Leray-1-Leu-127	25239	13611	13321	13321	13251	0.5250208

Supplementary Table S1: Read counts per sample through the DADA2 pipeline

**Leray-1-Leu-128**	**5083**	**74**	**58**	**58**	**58**	**0.01141058**
**Leray-1-Leu-129**	**5110**	**482**	**392**	**392**	**392**	**0.07671233**
**Leray-1-Leu-13**	**54842**	**33169**	**32660**	**32660**	**29332**	**0.53484556**
**Leray-1-Leu-130**	**10678**	**1137**	**904**	**904**	**836**	**0.07829181**
**Leray-1-Leu-131**	**6961**	**82**	**55**	**55**	**55**	**0.00790116**
**Leray-1-Leu-132**	**10422**	**1641**	**1583**	**1583**	**1583**	**0.15189023**
**Leray-1-Leu-133**	**5865**	**78**	**69**	**69**	**69**	**0.01176471**
**Leray-1-Leu-134**	**6789**	**537**	**456**	**456**	**456**	**0.06716748**
**Leray-1-Leu-135**	**6427**	**507**	**463**	**463**	**463**	**0.07203983**
**Leray-1-Leu-136**	**6205**	**276**	**172**	**172**	**172**	**0.02771958**
**Leray-1-Leu-137**	**3839**	**8**	**0**	**0**	**0**	**0**
**Leray-1-Leu-138**	**18800**	**5123**	**4802**	**4802**	**4696**	**0.24978723**
**Leray-1-Leu-139**	**7828**	**352**	**238**	**238**	**238**	**0.03040368**
**Leray-1-Leu-14**	**33628**	**15905**	**15685**	**15685**	**15676**	**0.46615915**
**Leray-1-Leu-140**	**7257**	**567**	**476**	**476**	**476**	**0.06559184**
**Leray-1-Leu-141**	**5563**	**162**	**131**	**131**	**131**	**0.02354845**
**Leray-1-Leu-142**	**3374**	**370**	**191**	**191**	**191**	**0.05660937**
**Leray-1-Leu-143**	**6578**	**219**	**182**	**182**	**182**	**0.02766798**
**Leray-1-Leu-144**	**16536**	**2029**	**1817**	**1817**	**1670**	**0.10099178**
**Leray-1-Leu-145**	**11568**	**2582**	**2499**	**2499**	**2499**	**0.21602697**
**Leray-1-Leu-146**	**6570**	**40**	**0**	**0**	**0**	**0**
**Leray-1-Leu-147**	**12397**	**682**	**642**	**642**	**642**	**0.05178672**
**Leray-1-Leu-148**	**13497**	**1320**	**1173**	**1173**	**1173**	**0.0869082**
**Leray-1-Leu-149**	**21111**	**7465**	**7237**	**7237**	**7237**	**0.34280707**
**Leray-1-Leu-15**	**46716**	**24472**	**23231**	**23231**	**19357**	**0.41435482**
**Leray-1-Leu-150**	**6666**	**55**	**25**	**25**	**25**	**0.00375038**
**Leray-1-Leu-151**	**12564**	**4902**	**4657**	**4657**	**4021**	**0.32004139**
**Leray-1-Leu-152**	**3950**	**14**	**4**	**4**	**4**	**0.00101266**
**Leray-1-Leu-153**	**31154**	**11570**	**11086**	**11086**	**10047**	**0.3224947**
**Leray-1-Leu-154**	**24974**	**7400**	**7004**	**7004**	**6823**	**0.27320413**
**Leray-1-Leu-155**	**8500**	**35**	**0**	**0**	**0**	**0**
**Leray-1-Leu-156**	**55261**	**28251**	**27500**	**27500**	**23577**	**0.42664809**
**Leray-1-Leu-157**	**5304**	**77**	**30**	**30**	**30**	**0.00565611**
**Leray-1-Leu-158**	**11680**	**1413**	**1366**	**1366**	**1366**	**0.11695205**
**Leray-1-Leu-159**	**11448**	**3522**	**3452**	**3452**	**3291**	**0.28747379**
**Leray-1-Leu-16**	**5109**	**276**	**237**	**237**	**237**	**0.04638873**
**Leray-1-Leu-160**	**4304**	**43**	**29**	**29**	**29**	**0.00673792**
**Leray-1-Leu-161**	**40544**	**34590**	**33504**	**33504**	**29518**	**0.72804854**
**Leray-1-Leu-162**	**38507**	**19936**	**19611**	**19611**	**19517**	**0.50684291**

Supplementary Table S1: Read counts per sample through the DADA2 pipeline

**Leray-1-Leu-163**	**10051**	**1580**	**1501**	**1501**	**1501**	**0.14933837**
**Leray-1-Leu-164**	**28349**	**8825**	**8060**	**8060**	**7294**	**0.25729303**
**Leray-1-Leu-165**	**18332**	**2871**	**2743**	**2743**	**2607**	**0.14221034**
**Leray-1-Leu-166**	**17883**	**7567**	**7402**	**7402**	**7372**	**0.41223508**
**Leray-1-Leu-167**	**4200**	**121**	**68**	**68**	**68**	**0.01619048**
**Leray-1-Leu-168**	**4907**	**293**	**179**	**179**	**179**	**0.0364785**
**Leray-1-Leu-169**	**10614**	**24**	**0**	**0**	**0**	**0**
**Leray-1-Leu-17**	**5045**	**21**	**0**	**0**	**0**	**0**
**Leray-1-Leu-170**	**14406**	**6572**	**6326**	**6326**	**5947**	**0.41281411**
**Leray-1-Leu-171**	**5114**	**192**	**156**	**156**	**156**	**0.0305045**
**Leray-1-Leu-172**	**5484**	**224**	**207**	**207**	**207**	**0.03774617**
**Leray-1-Leu-173**	**5717**	**579**	**552**	**552**	**552**	**0.09655414**
**Leray-1-Leu-174**	**4057**	**425**	**299**	**299**	**299**	**0.07369978**
**Leray-1-Leu-175**	**10260**	**96**	**36**	**36**	**36**	**0.00350877**
**Leray-1-Leu-176**	**4299**	**25**	**8**	**8**	**8**	**0.0018609**
**Leray-1-Leu-177**	**4277**	**18**	**0**	**0**	**0**	**0**
**Leray-1-Leu-178**	**23495**	**7077**	**6984**	**6984**	**6984**	**0.29725474**
**Leray-1-Leu-179**	**9072**	**1545**	**1515**	**1515**	**1515**	**0.16699735**
**Leray-1-Leu-18**	**25209**	**8271**	**8063**	**8063**	**8063**	**0.31984609**
**Leray-1-Leu-180**	**3709**	**373**	**355**	**355**	**355**	**0.09571313**
**Leray-1-Leu-181**	**40058**	**36469**	**36144**	**36144**	**35980**	**0.89819761**
**Leray-1-Leu-182**	**39165**	**30572**	**29509**	**29509**	**24444**	**0.62412869**
**Leray-1-Leu-183**	**21155**	**8311**	**8135**	**8135**	**7790**	**0.36823446**
**Leray-1-Leu-184**	**20765**	**3964**	**2386**	**2386**	**2121**	**0.10214303**
**Leray-1-Leu-185**	**4425**	**56**	**16**	**16**	**16**	**0.00361582**
**Leray-1-Leu-186**	**14892**	**2182**	**2046**	**2046**	**2001**	**0.13436745**
**Leray-1-Leu-187**	**5478**	**321**	**251**	**251**	**251**	**0.04581964**
**Leray-1-Leu-188**	**32338**	**14446**	**14220**	**14220**	**14204**	**0.43923557**
**Leray-1-Leu-189**	**6892**	**364**	**310**	**310**	**310**	**0.04497969**
**Leray-1-Leu-19**	**47076**	**25270**	**24274**	**24274**	**18949**	**0.40251933**
**Leray-1-Leu-190**	**8013**	**256**	**213**	**213**	**213**	**0.0265818**
**Leray-1-Leu-191**	**4369**	**643**	**528**	**528**	**528**	**0.12085145**
**Leray-1-Leu-192**	**15154**	**1381**	**1262**	**1262**	**1193**	**0.07872509**
**Leray-1-Leu-193**	**32022**	**14015**	**13809**	**13809**	**13045**	**0.40737618**
**Leray-1-Leu-194**	**4879**	**485**	**361**	**361**	**361**	**0.07399057**
**Leray-1-Leu-195**	**48702**	**30019**	**29347**	**29347**	**28173**	**0.57847727**
**Leray-1-Leu-196**	**8059**	**1677**	**1620**	**1620**	**1609**	**0.19965256**
**Leray-1-Leu-197**	**4867**	**362**	**334**	**334**	**334**	**0.06862544**
**Leray-1-Leu-198**	**6596**	**782**	**697**	**697**	**697**	**0.1056701**

Supplementary Table S1: Read counts per sample through the DADA2 pipeline

**Leray-1-Leu-199**	**10369**	**9176**	**7528**	**7528**	**5950**	**0.57382583**
**Leray-1-Leu-2**	**6078**	**54**	**23**	**23**	**23**	**0.00378414**
**Leray-1-Leu-20**	**8232**	**769**	**746**	**746**	**746**	**0.09062196**
**Leray-1-Leu-200**	**30989**	**27862**	**27190**	**27190**	**20534**	**0.66262222**
**Leray-1-Leu-201**	**36764**	**30524**	**29473**	**29473**	**27541**	**0.74912958**
**Leray-1-Leu-202**	**42447**	**37052**	**35734**	**35734**	**27803**	**0.65500507**
**Leray-1-Leu-203**	**55070**	**50731**	**48530**	**48530**	**31642**	**0.57457781**
**Leray-1-Leu-204**	**6213**	**1707**	**1360**	**1360**	**1360**	**0.21889586**
**Leray-1-Leu-205**	**8678**	**1449**	**1392**	**1392**	**1392**	**0.16040562**
**Leray-1-Leu-206**	**21050**	**7122**	**6766**	**6766**	**6308**	**0.29966746**
**Leray-1-Leu-207**	**33151**	**27391**	**26615**	**26615**	**22492**	**0.67847124**
**Leray-1-Leu-208**	**1409**	**206**	**169**	**169**	**169**	**0.11994322**
**Leray-1-Leu-209**	**48585**	**38122**	**36733**	**36733**	**25176**	**0.51818462**
**Leray-1-Leu-21**	**54418**	**29265**	**27930**	**27930**	**23571**	**0.43314712**
**Leray-1-Leu-210**	**12853**	**4925**	**4848**	**4848**	**4631**	**0.36030499**
**Leray-1-Leu-211**	**38888**	**25904**	**25004**	**25004**	**21321**	**0.54826682**
**Leray-1-Leu-212**	**37417**	**17897**	**16976**	**16976**	**16666**	**0.44541251**
**Leray-1-Leu-213**	**39144**	**32731**	**31397**	**31397**	**26277**	**0.67129062**
**Leray-1-Leu-214**	**59384**	**34024**	**33575**	**33575**	**33247**	**0.55986461**
**Leray-1-Leu-215**	**8565**	**6221**	**5803**	**5803**	**5461**	**0.63759486**
**Leray-1-Leu-216**	**2955**	**992**	**935**	**935**	**935**	**0.31641286**
**Leray-1-Leu-217**	**3780**	**10**	**0**	**0**	**0**	**0**
**Leray-1-Leu-218**	**449**	**148**	**99**	**99**	**99**	**0.22048998**
**Leray-1-Leu-219**	**61452**	**31995**	**30428**	**30428**	**27664**	**0.45017249**
**Leray-1-Leu-22**	**40214**	**16349**	**15859**	**15859**	**14596**	**0.36295817**
**Leray-1-Leu-220**	**42280**	**38130**	**36576**	**36576**	**29015**	**0.68625828**
**Leray-1-Leu-221**	**6853**	**3106**	**3019**	**3019**	**3019**	**0.44053699**
**Leray-1-Leu-222**	**6027**	**686**	**666**	**666**	**666**	**0.11050274**
**Leray-1-Leu-223**	**6686**	**857**	**705**	**705**	**705**	**0.10544421**
**Leray-1-Leu-224**	**3469**	**410**	**313**	**313**	**297**	**0.08561545**
**Leray-1-Leu-225**	**29439**	**17224**	**16835**	**16835**	**14885**	**0.50562179**
**Leray-1-Leu-226**	**6560**	**528**	**388**	**388**	**371**	**0.05655488**
**Leray-1-Leu-227**	**41650**	**30457**	**30046**	**30046**	**29954**	**0.71918367**
**Leray-1-Leu-228**	**5482**	**82**	**40**	**40**	**40**	**0.00729661**
**Leray-1-Leu-229**	**15257**	**5169**	**4929**	**4929**	**4785**	**0.31362653**
**Leray-1-Leu-23**	**45224**	**27870**	**26926**	**26926**	**24571**	**0.54331771**
**Leray-1-Leu-230**	**5053**	**1007**	**916**	**916**	**916**	**0.18127845**
**Leray-1-Leu-231**	**3754**	**373**	**301**	**301**	**301**	**0.08018114**
**Leray-1-Leu-232**	**2632**	**3**	**0**	**0**	**0**	**0**

Supplementary Table S1: Read counts per sample through the DADA2 pipeline

**Leray-1-Leu-233**	**5578**	**11**	**0**	**0**	**0**	**0**
**Leray-1-Leu-234**	**44639**	**37236**	**33760**	**33760**	**17328**	**0.38818074**
**Leray-1-Leu-235**	**7388**	**840**	**781**	**781**	**781**	**0.10571197**
**Leray-1-Leu-236**	**16129**	**1527**	**1251**	**1251**	**1251**	**0.07756216**
**Leray-1-Leu-237**	**14283**	**13058**	**12179**	**12179**	**12156**	**0.85108171**
**Leray-1-Leu-238**	**4890**	**544**	**537**	**537**	**537**	**0.10981595**
**Leray-1-Leu-239**	**101**	**18**	**0**	**0**	**0**	**0**
**Leray-1-Leu-24**	**41838**	**18051**	**16984**	**16984**	**14939**	**0.35706774**
**Leray-1-Leu-240**	**3709**	**19**	**0**	**0**	**0**	**0**
**Leray-1-Leu-241**	**32564**	**20028**	**19151**	**19151**	**17119**	**0.52570323**
**Leray-1-Leu-242**	**14311**	**4506**	**4420**	**4420**	**4214**	**0.29445881**
**Leray-1-Leu-243**	**34941**	**18253**	**17447**	**17447**	**16494**	**0.47205289**
**Leray-1-Leu-244**	**33323**	**9527**	**8864**	**8864**	**8763**	**0.26297152**
**Leray-1-Leu-245**	**22201**	**11010**	**10436**	**10436**	**9456**	**0.42592676**
**Leray-1-Leu-246**	**141**	**41**	**24**	**24**	**24**	**0.17021277**
**Leray-1-Leu-247**	**6645**	**647**	**534**	**534**	**534**	**0.08036117**
**Leray-1-Leu-248**	**4384**	**84**	**59**	**59**	**59**	**0.01345803**
**Leray-1-Leu-249**	**38246**	**27728**	**26906**	**26906**	**25181**	**0.65839565**
**Leray-1-Leu-25**	**3855**	**377**	**315**	**315**	**315**	**0.08171206**
**Leray-1-Leu-250**	**6849**	**709**	**665**	**665**	**665**	**0.09709447**
**Leray-1-Leu-251**	**4441**	**398**	**365**	**365**	**365**	**0.0821887**
**Leray-1-Leu-252**	**33294**	**18976**	**18747**	**18747**	**18410**	**0.55295248**
**Leray-1-Leu-253**	**14437**	**4702**	**4516**	**4516**	**4516**	**0.31280737**
**Leray-1-Leu-254**	**6238**	**433**	**131**	**131**	**131**	**0.02100032**
**Leray-1-Leu-255**	**23982**	**9266**	**8519**	**8519**	**7618**	**0.31765491**
**Leray-1-Leu-256**	**34661**	**14663**	**14358**	**14358**	**14246**	**0.41100949**
**Leray-1-Leu-257**	**28487**	**14810**	**14191**	**14191**	**14018**	**0.49208411**
**Leray-1-Leu-258**	**42984**	**17007**	**16924**	**16924**	**16924**	**0.3937279**
**Leray-1-Leu-259**	**21401**	**8398**	**8287**	**8287**	**8240**	**0.38502874**
**Leray-1-Leu-26**	**6786**	**10**	**0**	**0**	**0**	**0**
**Leray-1-Leu-260**	**12385**	**1934**	**1833**	**1833**	**1833**	**0.14800161**
**Leray-1-Leu-261**	**34650**	**24180**	**23779**	**23779**	**23779**	**0.68626263**
**Leray-1-Leu-262**	**6772**	**201**	**171**	**171**	**171**	**0.02525103**
**Leray-1-Leu-263**	**8904**	**2229**	**2190**	**2190**	**2190**	**0.24595687**
**Leray-1-Leu-264**	**37263**	**15472**	**14749**	**14749**	**12479**	**0.33488984**
**Leray-1-Leu-265**	**5157**	**36**	**17**	**17**	**17**	**0.00329649**
**Leray-1-Leu-266**	**3818**	**87**	**31**	**31**	**31**	**0.00811943**
**Leray-1-Leu-267**	**6167**	**59**	**46**	**46**	**46**	**0.00745906**
**Leray-1-Leu-268**	**21220**	**7458**	**7138**	**7138**	**6959**	**0.32794533**

Supplementary Table S1: Read counts per sample through the DADA2 pipeline

**Leray-1-Leu-269**	**32082**	**18200**	**17977**	**17977**	**17285**	**0.53877564**
**Leray-1-Leu-27**	**6989**	**834**	**815**	**815**	**815**	**0.11661182**
**Leray-1-Leu-270**	**12326**	**5253**	**4945**	**4945**	**4619**	**0.37473633**
**Leray-1-Leu-271**	**5273**	**178**	**143**	**143**	**143**	**0.02711929**
**Leray-1-Leu-272**	**15578**	**2372**	**2015**	**2015**	**2015**	**0.12934908**
**Leray-1-Leu-273**	**4305**	**51**	**34**	**34**	**34**	**0.00789779**
**Leray-1-Leu-274**	**4626**	**192**	**137**	**137**	**137**	**0.02961522**
**Leray-1-Leu-275**	**3237**	**42**	**27**	**27**	**27**	**0.00834106**
**Leray-1-Leu-276**	**4322**	**169**	**128**	**128**	**128**	**0.02961592**
**Leray-1-Leu-277**	**14340**	**6069**	**5909**	**5909**	**5872**	**0.40948396**
**Leray-1-Leu-278**	**2435**	**54**	**29**	**29**	**29**	**0.01190965**
**Leray-1-Leu-279**	**5109**	**84**	**75**	**75**	**75**	**0.01467998**
**Leray-1-Leu-28**	**7828**	**1019**	**992**	**992**	**959**	**0.12250894**
**Leray-1-Leu-280**	**27027**	**10651**	**10426**	**10426**	**10396**	**0.38465238**
**Leray-1-Leu-281**	**11365**	**2691**	**2484**	**2484**	**2484**	**0.21856577**
**Leray-1-Leu-282**	**30839**	**12328**	**11594**	**11594**	**10906**	**0.35364311**
**Leray-1-Leu-283**	**3584**	**168**	**151**	**151**	**151**	**0.0421317**
**Leray-1-Leu-284**	**30477**	**11920**	**11706**	**11706**	**10682**	**0.35049382**
**Leray-1-Leu-285**	**6899**	**153**	**138**	**138**	**138**	**0.0200029**
**Leray-1-Leu-286**	**2253**	**66**	**58**	**58**	**58**	**0.02574345**
**Leray-1-Leu-287**	**4086**	**30**	**11**	**11**	**11**	**0.00269212**
**Leray-1-Leu-288**	**7564**	**1227**	**1207**	**1207**	**1207**	**0.15957166**
**Leray-1-Leu-29**	**10988**	**1102**	**1052**	**1052**	**1052**	**0.09574081**
**Leray-1-Leu-3**	**6564**	**568**	**540**	**540**	**540**	**0.08226691**
**Leray-1-Leu-30**	**10774**	**1360**	**1265**	**1265**	**1265**	**0.11741229**
**Leray-1-Leu-31**	**5320**	**80**	**58**	**58**	**58**	**0.01090226**
**Leray-1-Leu-32**	**9253**	**49**	**31**	**31**	**31**	**0.00335026**
**Leray-1-Leu-33**	**12442**	**239**	**146**	**146**	**146**	**0.01173445**
**Leray-1-Leu-34**	**14894**	**3924**	**3553**	**3553**	**3553**	**0.23855244**
**Leray-1-Leu-35**	**4411**	**122**	**107**	**107**	**107**	**0.02425754**
**Leray-1-Leu-36**	**12449**	**504**	**467**	**467**	**467**	**0.03751305**
**Leray-1-Leu-37**	**7796**	**1009**	**981**	**981**	**981**	**0.12583376**
**Leray-1-Leu-38**	**7946**	**342**	**314**	**314**	**314**	**0.03951674**
**Leray-1-Leu-39**	**5012**	**267**	**193**	**193**	**193**	**0.03850758**
**Leray-1-Leu-4**	**16306**	**49**	**15**	**15**	**15**	**0.00091991**
**Leray-1-Leu-40**	**14239**	**152**	**101**	**101**	**101**	**0.00709319**
**Leray-1-Leu-41**	**14762**	**1366**	**770**	**770**	**770**	**0.05216095**
**Leray-1-Leu-42**	**15614**	**37**	**14**	**14**	**14**	**0.00089663**
**Leray-1-Leu-43**	**7323**	**580**	**550**	**550**	**550**	**0.07510583**

Supplementary Table S1: Read counts per sample through the DADA2 pipeline

**Leray-1-Leu-44**	**9539**	**1257**	**1181**	**1181**	**1181**	**0.12380753**
**Leray-1-Leu-45**	**9096**	**1681**	**1583**	**1583**	**1583**	**0.17403254**
**Leray-1-Leu-46**	**9478**	**987**	**925**	**925**	**925**	**0.09759443**
**Leray-1-Leu-47**	**6764**	**1023**	**934**	**934**	**934**	**0.13808397**
**Leray-1-Leu-48**	**6682**	**120**	**95**	**95**	**95**	**0.0142173**
**Leray-1-Leu-5**	**38586**	**17414**	**16548**	**16548**	**15209**	**0.3941585**
**Leray-1-Leu-6**	**25048**	**7439**	**7233**	**7233**	**7225**	**0.28844618**
**Leray-1-Leu-7**	**6716**	**231**	**154**	**154**	**154**	**0.02293032**
**Leray-1-Leu-8**	**3037**	**87**	**55**	**55**	**55**	**0.01810998**
**Leray-1-Leu-9**	**4394**	**31**	**12**	**12**	**12**	**0.002731**
**Leray-1-Leu-97**	**11396**	**123**	**70**	**70**	**70**	**0.00614251**
**Leray-1-Leu-98**	**21751**	**4063**	**3872**	**3872**	**3843**	**0.17668153**
**Leray-1-Leu-99**	**8394**	**918**	**834**	**834**	**834**	**0.09935668**
**Leray-1-Leu-Neg1**	**4830**	**488**	**304**	**304**	**303**	**0.06273292**
**Leray-1-Leu-Neg2**	**2936**	**410**	**223**	**223**	**223**	**0.07595368**
**Leray-1-Leu-Neg3**	**4733**	**229**	**209**	**209**	**209**	**0.04415804**
**Leray-1-Mock-com-2**	**34574**	**33106**	**32190**	**32190**	**27204**	**0.78683404**
**Leray-2-FWM-10**	**12222**	**11319**	**11296**	**11296**	**11296**	**0.92423499**
**Leray-2-FWM-2**	**42634**	**40921**	**39787**	**39787**	**39066**	**0.91631093**
**Leray-2-FWM-3**	**49045**	**43678**	**38231**	**38231**	**34954**	**0.71269243**
**Leray-2-FWM-6**	**106**	**62**	**33**	**33**	**33**	**0.31132075**
**Leray-2-FWM-7**	**38229**	**36922**	**36821**	**36821**	**36821**	**0.96316932**
**Leray-2-FWM-9**	**17065**	**16149**	**15838**	**15838**	**15838**	**0.92809845**
**Leray-2-Leu-1**	**16195**	**15587**	**14184**	**14184**	**8833**	**0.54541525**
**Leray-2-Leu-10**	**13234**	**12669**	**11652**	**11652**	**8141**	**0.61515793**
**Leray-2-Leu-100**	**16240**	**15554**	**12983**	**12983**	**4842**	**0.29815271**
**Leray-2-Leu-101**	**13437**	**12553**	**12261**	**12261**	**6310**	**0.46959887**
**Leray-2-Leu-102**	**20927**	**18436**	**4164**	**4164**	**2530**	**0.12089645**
**Leray-2-Leu-103**	**15950**	**11848**	**8492**	**8492**	**4385**	**0.27492163**
**Leray-2-Leu-104**	**9064**	**8648**	**7560**	**7560**	**2906**	**0.320609**
**Leray-2-Leu-105**	**15804**	**14477**	**12802**	**12802**	**6535**	**0.41350291**
**Leray-2-Leu-106**	**4377**	**2429**	**2281**	**2281**	**1519**	**0.34704135**
**Leray-2-Leu-107**	**217**	**105**	**87**	**87**	**87**	**0.40092166**
**Leray-2-Leu-108**	**334**	**262**	**189**	**189**	**189**	**0.56586826**
**Leray-2-Leu-109**	**59**	**13**	**0**	**0**	**0**	**0**
**Leray-2-Leu-11**	**12379**	**11896**	**10749**	**10749**	**4853**	**0.3920349**
**Leray-2-Leu-110**	**241**	**101**	**65**	**65**	**65**	**0.26970954**
**Leray-2-Leu-111**	**313**	**230**	**212**	**212**	**212**	**0.67731629**
**Leray-2-Leu-112**	**258**	**81**	**48**	**48**	**48**	**0.18604651**

Supplementary Table S1: Read counts per sample through the DADA2 pipeline

**Leray-2-Leu-113**	**84**	**56**	**55**	**55**	**55**	**0.6547619**
**Leray-2-Leu-114**	**156**	**59**	**42**	**42**	**42**	**0.26923077**
**Leray-2-Leu-115**	**89**	**25**	**17**	**17**	**17**	**0.19101124**
**Leray-2-Leu-116**	**148**	**43**	**0**	**0**	**0**	**0**
**Leray-2-Leu-117**	**48**	**4**	**0**	**0**	**0**	**0**
**Leray-2-Leu-118**	**63**	**13**	**0**	**0**	**0**	**0**
**Leray-2-Leu-119**	**265**	**217**	**168**	**168**	**168**	**0.63396226**
**Leray-2-Leu-12**	**12743**	**12136**	**10302**	**10302**	**5007**	**0.3929216**
**Leray-2-Leu-120**	**46**	**5**	**0**	**0**	**0**	**0**
**Leray-2-Leu-121**	**131**	**34**	**30**	**30**	**30**	**0.22900763**
**Leray-2-Leu-122**	**35**	**6**	**0**	**0**	**0**	**0**
**Leray-2-Leu-123**	**125**	**64**	**0**	**0**	**0**	**0**
**Leray-2-Leu-124**	**155**	**57**	**23**	**23**	**23**	**0.1483871**
**Leray-2-Leu-125**	**100**	**15**	**8**	**8**	**8**	**0.08**
**Leray-2-Leu-126**	**24**	**6**	**0**	**0**	**0**	**0**
**Leray-2-Leu-127**	**215**	**69**	**22**	**22**	**22**	**0.10232558**
**Leray-2-Leu-128**	**61**	**4**	**0**	**0**	**0**	**0**
**Leray-2-Leu-129**	**45**	**8**	**0**	**0**	**0**	**0**
**Leray-2-Leu-13**	**15554**	**14486**	**13517**	**13517**	**6860**	**0.4410441**
**Leray-2-Leu-130**	**71**	**13**	**9**	**9**	**9**	**0.12676056**
**Leray-2-Leu-131**	**152**	**87**	**49**	**49**	**49**	**0.32236842**
**Leray-2-Leu-132**	**146**	**94**	**39**	**39**	**39**	**0.26712329**
**Leray-2-Leu-133**	**166**	**107**	**78**	**78**	**54**	**0.3253012**
**Leray-2-Leu-134**	**44**	**10**	**0**	**0**	**0**	**0**
**Leray-2-Leu-135**	**94**	**45**	**0**	**0**	**0**	**0**
**Leray-2-Leu-136**	**58**	**5**	**2**	**2**	**2**	**0.03448276**
**Leray-2-Leu-137**	**27**	**3**	**2**	**2**	**2**	**0.07407407**
**Leray-2-Leu-138**	**136**	**40**	**25**	**25**	**25**	**0.18382353**
**Leray-2-Leu-139**	**237**	**176**	**0**	**0**	**0**	**0**
**Leray-2-Leu-14**	**11716**	**11040**	**10486**	**10486**	**5787**	**0.49393991**
**Leray-2-Leu-140**	**217**	**158**	**113**	**113**	**113**	**0.52073733**
**Leray-2-Leu-141**	**234**	**174**	**129**	**129**	**129**	**0.55128205**
**Leray-2-Leu-142**	**44**	**18**	**0**	**0**	**0**	**0**
**Leray-2-Leu-143**	**40**	**8**	**0**	**0**	**0**	**0**
**Leray-2-Leu-144**	**20332**	**8999**	**8728**	**8728**	**7384**	**0.36317136**
**Leray-2-Leu-145**	**14084**	**524**	**131**	**131**	**131**	**0.00930133**
**Leray-2-Leu-146**	**5317**	**372**	**347**	**347**	**347**	**0.06526237**
**Leray-2-Leu-147**	**10578**	**2101**	**1998**	**1998**	**1998**	**0.18888259**
**Leray-2-Leu-148**	**8671**	**2071**	**1852**	**1852**	**1852**	**0.21358551**

Supplementary Table S1: Read counts per sample through the DADA2 pipeline

**Leray-2-Leu-149**	**29776**	**11299**	**10558**	**10558**	**10483**	**0.35206206**
**Leray-2-Leu-15**	**12900**	**12093**	**10057**	**10057**	**5883**	**0.45604651**
**Leray-2-Leu-150**	**8681**	**930**	**793**	**793**	**793**	**0.09134892**
**Leray-2-Leu-151**	**11707**	**5467**	**5146**	**5146**	**4497**	**0.38412915**
**Leray-2-Leu-152**	**15445**	**96**	**9**	**9**	**9**	**0.00058271**
**Leray-2-Leu-153**	**47086**	**30018**	**27807**	**27807**	**22868**	**0.48566453**
**Leray-2-Leu-154**	**24936**	**10563**	**10148**	**10148**	**9360**	**0.37536092**
**Leray-2-Leu-155**	**20639**	**743**	**181**	**181**	**180**	**0.00872135**
**Leray-2-Leu-156**	**40172**	**31683**	**30898**	**30898**	**26109**	**0.6499303**
**Leray-2-Leu-157**	**40961**	**23974**	**23530**	**23530**	**21968**	**0.53631503**
**Leray-2-Leu-158**	**14051**	**7706**	**7606**	**7606**	**7409**	**0.52729343**
**Leray-2-Leu-159**	**18285**	**33**	**0**	**0**	**0**	**0**
**Leray-2-Leu-16**	**13323**	**12784**	**11432**	**11432**	**6995**	**0.5250319**
**Leray-2-Leu-160**	**14759**	**119**	**21**	**21**	**21**	**0.00142286**
**Leray-2-Leu-161**	**35471**	**31409**	**30676**	**30676**	**27030**	**0.76203095**
**Leray-2-Leu-162**	**37800**	**27814**	**27382**	**27382**	**27131**	**0.71775132**
**Leray-2-Leu-163**	**17876**	**5458**	**5305**	**5305**	**5255**	**0.29396957**
**Leray-2-Leu-164**	**28388**	**11439**	**11103**	**11103**	**10712**	**0.37734254**
**Leray-2-Leu-165**	**30460**	**18304**	**17727**	**17727**	**13647**	**0.4480302**
**Leray-2-Leu-166**	**19993**	**7442**	**7067**	**7067**	**7020**	**0.35112289**
**Leray-2-Leu-167**	**8258**	**473**	**432**	**432**	**432**	**0.05231291**
**Leray-2-Leu-168**	**8280**	**402**	**322**	**322**	**322**	**0.03888889**
**Leray-2-Leu-169**	**3616**	**15**	**0**	**0**	**0**	**0**
**Leray-2-Leu-17**	**16331**	**15700**	**14389**	**14389**	**5557**	**0.3402731**
**Leray-2-Leu-170**	**18560**	**7908**	**7427**	**7427**	**6988**	**0.37650862**
**Leray-2-Leu-171**	**6865**	**477**	**367**	**367**	**367**	**0.05345958**
**Leray-2-Leu-172**	**5302**	**127**	**117**	**117**	**117**	**0.02206714**
**Leray-2-Leu-173**	**6459**	**1340**	**1304**	**1304**	**1304**	**0.20188884**
**Leray-2-Leu-174**	**4095**	**356**	**248**	**248**	**248**	**0.06056166**
**Leray-2-Leu-175**	**13627**	**633**	**537**	**537**	**537**	**0.03940706**
**Leray-2-Leu-176**	**20104**	**62**	**0**	**0**	**0**	**0**
**Leray-2-Leu-177**	**4497**	**12**	**0**	**0**	**0**	**0**
**Leray-2-Leu-178**	**16183**	**5074**	**5017**	**5017**	**5017**	**0.31001668**
**Leray-2-Leu-179**	**5507**	**826**	**801**	**801**	**801**	**0.14545124**
**Leray-2-Leu-18**	**14237**	**13617**	**11351**	**11351**	**6208**	**0.43604692**
**Leray-2-Leu-180**	**4498**	**54**	**30**	**30**	**30**	**0.00666963**
**Leray-2-Leu-181**	**36566**	**32515**	**32274**	**32274**	**32141**	**0.87898594**
**Leray-2-Leu-182**	**20689**	**17839**	**16988**	**16988**	**14640**	**0.70762241**
**Leray-2-Leu-183**	**4372**	**1786**	**1759**	**1759**	**1729**	**0.39547118**

Supplementary Table S1: Read counts per sample through the DADA2 pipeline

**Leray-2-Leu-184**	**4647**	**205**	**195**	**195**	**195**	**0.04196256**
**Leray-2-Leu-185**	**3843**	**19**	**0**	**0**	**0**	**0**
**Leray-2-Leu-186**	**19054**	**6293**	**6176**	**6176**	**6153**	**0.32292432**
**Leray-2-Leu-187**	**4898**	**169**	**111**	**111**	**111**	**0.02266231**
**Leray-2-Leu-188**	**10464**	**2177**	**2141**	**2141**	**2099**	**0.20059251**
**Leray-2-Leu-189**	**5404**	**30**	**0**	**0**	**0**	**0**
**Leray-2-Leu-19**	**13599**	**13006**	**11816**	**11816**	**6766**	**0.49753658**
**Leray-2-Leu-190**	**5477**	**663**	**617**	**617**	**617**	**0.11265291**
**Leray-2-Leu-191**	**12356**	**564**	**82**	**82**	**82**	**0.00663645**
**Leray-2-Leu-192**	**16988**	**6232**	**6013**	**6013**	**5541**	**0.32617142**
**Leray-2-Leu-193**	**22440**	**8634**	**8347**	**8347**	**7772**	**0.34634581**
**Leray-2-Leu-194**	**4596**	**526**	**416**	**416**	**416**	**0.09051349**
**Leray-2-Leu-195**	**34378**	**17641**	**17192**	**17192**	**16541**	**0.48115074**
**Leray-2-Leu-196**	**4588**	**219**	**143**	**143**	**143**	**0.03116827**
**Leray-2-Leu-197**	**7081**	**1006**	**843**	**843**	**843**	**0.11905098**
**Leray-2-Leu-198**	**5200**	**254**	**232**	**232**	**232**	**0.04461538**
**Leray-2-Leu-199**	**9442**	**6058**	**4958**	**4958**	**4257**	**0.45085787**
**Leray-2-Leu-2**	**13766**	**13237**	**12475**	**12475**	**7412**	**0.53842801**
**Leray-2-Leu-20**	**14829**	**14180**	**13772**	**13772**	**6601**	**0.44514128**
**Leray-2-Leu-200**	**29768**	**26675**	**26031**	**26031**	**19446**	**0.65325181**
**Leray-2-Leu-201**	**31974**	**27335**	**26391**	**26391**	**24388**	**0.76274473**
**Leray-2-Leu-202**	**40285**	**34924**	**33545**	**33545**	**26063**	**0.64696537**
**Leray-2-Leu-203**	**49032**	**43956**	**41664**	**41664**	**26135**	**0.53301925**
**Leray-2-Leu-204**	**18024**	**9373**	**9100**	**9100**	**8546**	**0.47414558**
**Leray-2-Leu-205**	**6048**	**1043**	**1009**	**1009**	**1009**	**0.16683201**
**Leray-2-Leu-206**	**16785**	**5469**	**5205**	**5205**	**4900**	**0.29192732**
**Leray-2-Leu-207**	**35530**	**12495**	**11725**	**11725**	**10402**	**0.29276668**
**Leray-2-Leu-208**	**12282**	**2944**	**2893**	**2893**	**2893**	**0.23554796**
**Leray-2-Leu-209**	**34851**	**30289**	**29035**	**29035**	**19801**	**0.5681616**
**Leray-2-Leu-21**	**14044**	**13324**	**12306**	**12306**	**7447**	**0.53026203**
**Leray-2-Leu-210**	**5122**	**477**	**453**	**453**	**453**	**0.08844201**
**Leray-2-Leu-211**	**40645**	**21480**	**20675**	**20675**	**16906**	**0.41594292**
**Leray-2-Leu-212**	**14208**	**2164**	**2009**	**2009**	**2009**	**0.14139921**
**Leray-2-Leu-213**	**36386**	**30847**	**29856**	**29856**	**24539**	**0.67440774**
**Leray-2-Leu-214**	**45629**	**35818**	**35411**	**35411**	**35099**	**0.76922571**
**Leray-2-Leu-215**	**18140**	**9915**	**9512**	**9512**	**9070**	**0.5**
**Leray-2-Leu-216**	**15214**	**4295**	**4239**	**4239**	**4234**	**0.27829631**
**Leray-2-Leu-217**	**5258**	**284**	**278**	**278**	**278**	**0.05287181**
**Leray-2-Leu-218**	**41980**	**33570**	**32261**	**32261**	**29340**	**0.69890424**

Supplementary Table S1: Read counts per sample through the DADA2 pipeline

**Leray-2-Leu-219**	**32900**	**15318**	**14656**	**14656**	**13784**	**0.41896657**
**Leray-2-Leu-22**	**14403**	**13764**	**11380**	**11380**	**7295**	**0.5064917**
**Leray-2-Leu-220**	**36631**	**32016**	**30708**	**30708**	**24275**	**0.66269007**
**Leray-2-Leu-221**	**12393**	**4849**	**4686**	**4686**	**4663**	**0.37626079**
**Leray-2-Leu-222**	**4947**	**409**	**402**	**402**	**402**	**0.08126137**
**Leray-2-Leu-223**	**7326**	**173**	**158**	**158**	**158**	**0.02156702**
**Leray-2-Leu-224**	**6400**	**323**	**204**	**204**	**204**	**0.031875**
**Leray-2-Leu-225**	**26662**	**17073**	**16385**	**16385**	**14076**	**0.52794239**
**Leray-2-Leu-226**	**5712**	**315**	**216**	**216**	**216**	**0.03781513**
**Leray-2-Leu-227**	**36958**	**25455**	**25181**	**25181**	**25181**	**0.68134098**
**Leray-2-Leu-228**	**4410**	**94**	**67**	**67**	**67**	**0.01519274**
**Leray-2-Leu-229**	**6157**	**2910**	**2636**	**2636**	**2636**	**0.42813058**
**Leray-2-Leu-23**	**13790**	**12894**	**12580**	**12580**	**7128**	**0.5168963**
**Leray-2-Leu-230**	**3925**	**73**	**61**	**61**	**61**	**0.0155414**
**Leray-2-Leu-231**	**5268**	**7**	**0**	**0**	**0**	**0**
**Leray-2-Leu-232**	**3457**	**5**	**0**	**0**	**0**	**0**
**Leray-2-Leu-233**	**9241**	**18**	**0**	**0**	**0**	**0**
**Leray-2-Leu-234**	**51426**	**31238**	**28576**	**28576**	**15550**	**0.30237623**
**Leray-2-Leu-235**	**5561**	**162**	**115**	**115**	**115**	**0.02067973**
**Leray-2-Leu-236**	**19356**	**268**	**221**	**221**	**221**	**0.01141765**
**Leray-2-Leu-237**	**9127**	**8380**	**7695**	**7695**	**7633**	**0.83630985**
**Leray-2-Leu-238**	**7348**	**7**	**0**	**0**	**0**	**0**
**Leray-2-Leu-239**	**2838**	**65**	**32**	**32**	**32**	**0.01127555**
**Leray-2-Leu-24**	**15818**	**12107**	**10871**	**10871**	**6998**	**0.44240738**
**Leray-2-Leu-240**	**4340**	**50**	**41**	**41**	**41**	**0.009447**
**Leray-2-Leu-241**	**27175**	**20043**	**18920**	**18920**	**16625**	**0.61177553**
**Leray-2-Leu-242**	**12260**	**2595**	**2362**	**2362**	**2320**	**0.18923328**
**Leray-2-Leu-243**	**30681**	**17078**	**16382**	**16382**	**15877**	**0.51748639**
**Leray-2-Leu-244**	**35065**	**15333**	**14805**	**14805**	**14488**	**0.41317553**
**Leray-2-Leu-245**	**28501**	**16058**	**15133**	**15133**	**13630**	**0.47822883**
**Leray-2-Leu-246**	**12804**	**1874**	**1469**	**1469**	**1346**	**0.1051234**
**Leray-2-Leu-247**	**10876**	**1374**	**1197**	**1197**	**1197**	**0.11005885**
**Leray-2-Leu-248**	**5779**	**39**	**30**	**30**	**30**	**0.00519121**
**Leray-2-Leu-249**	**36130**	**26826**	**26224**	**26224**	**24317**	**0.67304179**
**Leray-2-Leu-25**	**17670**	**16932**	**15672**	**15672**	**7606**	**0.43044709**
**Leray-2-Leu-250**	**4568**	**832**	**803**	**803**	**803**	**0.17578809**
**Leray-2-Leu-251**	**3928**	**288**	**250**	**250**	**250**	**0.06364562**
**Leray-2-Leu-252**	**29705**	**20762**	**20565**	**20565**	**20563**	**0.69224036**
**Leray-2-Leu-253**	**21188**	**11012**	**10537**	**10537**	**9998**	**0.47187087**

Supplementary Table S1: Read counts per sample through the DADA2 pipeline

**Leray-2-Leu-254**	**2443**	**66**	**53**	**53**	**53**	**0.02169464**
**Leray-2-Leu-255**	**20292**	**6518**	**6065**	**6065**	**5863**	**0.2889316**
**Leray-2-Leu-256**	**34111**	**19480**	**18779**	**18779**	**18484**	**0.54187799**
**Leray-2-Leu-257**	**29244**	**15258**	**15092**	**15092**	**15048**	**0.51456709**
**Leray-2-Leu-258**	**32279**	**25233**	**24882**	**24882**	**24882**	**0.77084172**
**Leray-2-Leu-259**	**21216**	**7136**	**6999**	**6999**	**6999**	**0.32989253**
**Leray-2-Leu-26**	**17020**	**16329**	**15037**	**15037**	**7485**	**0.43977673**
**Leray-2-Leu-260**	**16575**	**6737**	**6667**	**6667**	**6667**	**0.40223228**
**Leray-2-Leu-261**	**33779**	**27127**	**26748**	**26748**	**26713**	**0.79081678**
**Leray-2-Leu-262**	**9589**	**3275**	**3216**	**3216**	**3192**	**0.33288143**
**Leray-2-Leu-263**	**10280**	**3001**	**2923**	**2923**	**2923**	**0.28433852**
**Leray-2-Leu-264**	**33190**	**18555**	**17661**	**17661**	**14893**	**0.44871949**
**Leray-2-Leu-265**	**3747**	**269**	**259**	**259**	**259**	**0.06912196**
**Leray-2-Leu-266**	**2715**	**63**	**27**	**27**	**27**	**0.00994475**
**Leray-2-Leu-267**	**3028**	**216**	**204**	**204**	**204**	**0.0673712**
**Leray-2-Leu-268**	**22729**	**9025**	**8609**	**8609**	**8495**	**0.37375159**
**Leray-2-Leu-269**	**28771**	**19037**	**18823**	**18823**	**18274**	**0.63515345**
**Leray-2-Leu-27**	**12748**	**12041**	**10896**	**10896**	**6087**	**0.47748666**
**Leray-2-Leu-270**	**29902**	**19138**	**18625**	**18625**	**17191**	**0.57491138**
**Leray-2-Leu-271**	**13608**	**1973**	**1904**	**1904**	**1904**	**0.1399177**
**Leray-2-Leu-272**	**21298**	**9906**	**9487**	**9487**	**9487**	**0.44544089**
**Leray-2-Leu-273**	**7216**	**1797**	**1719**	**1719**	**1719**	**0.23822062**
**Leray-2-Leu-274**	**1728**	**150**	**128**	**128**	**128**	**0.07407407**
**Leray-2-Leu-275**	**2884**	**65**	**59**	**59**	**59**	**0.0204577**
**Leray-2-Leu-276**	**4510**	**1013**	**963**	**963**	**963**	**0.2135255**
**Leray-2-Leu-277**	**12158**	**9578**	**9105**	**9105**	**9105**	**0.74888962**
**Leray-2-Leu-278**	**17473**	**7990**	**7928**	**7928**	**7891**	**0.45161106**
**Leray-2-Leu-279**	**3008**	**16**	**0**	**0**	**0**	**0**
**Leray-2-Leu-28**	**13460**	**12649**	**12198**	**12198**	**7262**	**0.53952452**
**Leray-2-Leu-280**	**29932**	**18903**	**18537**	**18537**	**18153**	**0.60647468**
**Leray-2-Leu-281**	**6119**	**662**	**642**	**642**	**642**	**0.1049191**
**Leray-2-Leu-282**	**25125**	**9738**	**9104**	**9104**	**8494**	**0.33806965**
**Leray-2-Leu-283**	**2990**	**438**	**425**	**425**	**425**	**0.14214047**
**Leray-2-Leu-284**	**28870**	**19691**	**19243**	**19243**	**17645**	**0.61118808**
**Leray-2-Leu-285**	**1269**	**200**	**188**	**188**	**188**	**0.14814815**
**Leray-2-Leu-286**	**10386**	**486**	**152**	**152**	**152**	**0.01463509**
**Leray-2-Leu-287**	**3076**	**67**	**53**	**53**	**53**	**0.01723017**
**Leray-2-Leu-288**	**4707**	**494**	**471**	**471**	**471**	**0.10006373**
**Leray-2-Leu-29**	**11582**	**11064**	**10539**	**10539**	**5953**	**0.51398722**

Supplementary Table S1: Read counts per sample through the DADA2 pipeline

**Leray-2-Leu-3**	**12078**	**11592**	**9682**	**9682**	**6195**	**0.51291605**
**Leray-2-Leu-30**	**14712**	**14157**	**13085**	**13085**	**6119**	**0.41591898**
**Leray-2-Leu-31**	**13227**	**12704**	**12015**	**12015**	**6474**	**0.48945339**
**Leray-2-Leu-32**	**13887**	**13333**	**12596**	**12596**	**5426**	**0.39072514**
**Leray-2-Leu-33**	**17309**	**16675**	**15927**	**15927**	**9606**	**0.5549714**
**Leray-2-Leu-34**	**12627**	**12103**	**11088**	**11088**	**5944**	**0.47073731**
**Leray-2-Leu-35**	**12867**	**12256**	**10946**	**10946**	**6715**	**0.52187767**
**Leray-2-Leu-36**	**12687**	**12126**	**11073**	**11073**	**6737**	**0.531016**
**Leray-2-Leu-37**	**11507**	**10863**	**10213**	**10213**	**6465**	**0.56183193**
**Leray-2-Leu-38**	**14300**	**13781**	**13498**	**13498**	**7033**	**0.49181818**
**Leray-2-Leu-39**	**11859**	**11360**	**10483**	**10483**	**4794**	**0.40424994**
**Leray-2-Leu-4**	**14426**	**13886**	**11107**	**11107**	**6318**	**0.43795924**
**Leray-2-Leu-40**	**15848**	**15311**	**13977**	**13977**	**6742**	**0.42541646**
**Leray-2-Leu-41**	**8327**	**7988**	**7581**	**7581**	**5255**	**0.63107962**
**Leray-2-Leu-42**	**11325**	**10793**	**10366**	**10366**	**4628**	**0.40865342**
**Leray-2-Leu-43**	**14789**	**13999**	**13262**	**13262**	**8015**	**0.54195686**
**Leray-2-Leu-44**	**13316**	**12087**	**11340**	**11340**	**7067**	**0.53071493**
**Leray-2-Leu-45**	**13992**	**12130**	**11491**	**11491**	**8119**	**0.58026015**
**Leray-2-Leu-46**	**16801**	**16141**	**14034**	**14034**	**7041**	**0.4190822**
**Leray-2-Leu-47**	**14344**	**13658**	**12868**	**12868**	**7930**	**0.55284439**
**Leray-2-Leu-48**	**12299**	**11838**	**10632**	**10632**	**4474**	**0.36376941**
**Leray-2-Leu-5**	**15398**	**14675**	**13471**	**13471**	**5828**	**0.37849071**
**Leray-2-Leu-6**	**15577**	**14918**	**13626**	**13626**	**6630**	**0.42562753**
**Leray-2-Leu-7**	**12414**	**11864**	**11429**	**11429**	**5995**	**0.48292251**
**Leray-2-Leu-8**	**13589**	**12255**	**10398**	**10398**	**6597**	**0.48546619**
**Leray-2-Leu-9**	**14855**	**14274**	**12569**	**12569**	**7113**	**0.47882868**
**Leray-2-Leu-97**	**11157**	**10584**	**9915**	**9915**	**6642**	**0.59532132**
**Leray-2-Leu-98**	**12761**	**12169**	**10700**	**10700**	**7671**	**0.60112844**
**Leray-2-Leu-99**	**16666**	**16101**	**12929**	**12929**	**5591**	**0.33547342**
**Leray-2-Leu-Neg1**	**8**	**1**	**1**	**1**	**1**	**0.125**
**Leray-2-Leu-Neg2**	**2702**	**3**	**1**	**1**	**1**	**0.0003701**
**Leray-2-Leu-Neg3**	**1297**	**181**	**166**	**166**	**166**	**0.12798766**
**Leray-2-Mock-com-2**	**32822**	**31509**	**30811**	**30811**	**27285**	**0.83130218**
