# Supplementary material for: Metabarcoding prey DNA from fecal samples of adult dragonflies shows no predicted sex differences, and substantial inter-individual variation, in diets
Source: PeerJ. 2021 Dec 17;9:e12634. doi: 10.7717/peerj.12634 (PMC8686731; doi:10.7717/peerj.12634)
Supplement: Supplemental Information 2 [file peerj-09-12634-s002.pdf]

Supplementary Article 1 for

Metabarcoding prey DNA from fecal samples of adult dragonflies shows no predicted sex differences, and substantial inter-individual variation, in diets

André Morrill, Kari M. Kaunisto, Julia J Mlynarek, Ella Sippola, Eero J Vesterinen, and Mark R. Forbes

Corresponding author: André Morrill (andre_morrill@carleton.ca)

We prepared a mock community sample to be able to assess tag-jumping, a known phenomenon in the Illumina sequencing platform, as well as to interpret the quantitative aspects in the molecular data (summary in figure below). We chose six insects from four orders, representing potential prey diversity: Diptera: *Coenosia mollicula, Allocotocera pulchella*; Auchenorrhyncha: *Grypotes puncticollis*; Hymenoptera: *Phygadeuon sp. 1*; Lepidoptera: *Crambus heringiellus, Apamea remissa*. The specimens were collected using a Malaise trap (August 2017) on the island of Seili in Turku Archipelago located in the Baltic Sea. The samples were carefully surface-sterilised, and identified to the species level if possible. DNA was extracted and subsequently measured for concentration using Qubit Fluorometer. All the samples were DNA barcoded; that is, amplified using universal primer pair LCO1490/HCO2198 (Folmer et al., 1994). DNA extraction and PCR conditions followed Pilipenko *et al.* (2012). The resulting Sanger sequences were trimmed using software Geneious Prime (Kearse et al., 2012) and compared against BOLD reference database (Ratnasingham and Hebert, 2007) and GenBank using BLAST (Altschul et al., 1990) to confirm the species identification. All the sequence matched nearly 100% to the identified species, except *Phygadeuon* species (closest match *Phygadeuon variabilis*). These sequences were included in the local annotated database to be used in the final probabilistic taxonomic assignation of the sequence variants of the molecular dietary data. Then, all DNA samples were normalized into equal concentration, and an equimolar amount of DNA from each of the six mock species were combined to form a mock community sample. The mock community sample, as well as all the mock samples individually were included in the dietary analysis to account for tag jumping (sample cross-talk), repeatability, and quantitative aspects of the molecular data. All the mock species were observed in the mock community sample, as well as in the individual mock samples. We noticed that the cicadid *Grypotes puncticollis* sample seemed to contain a cicadellidae parasitic fly (Diptera: Pipunculidae, *Eudorylas terminalis* [BOLD:ADF4796]), which in our opinion indicates a sensitive analysis and deep enough sequencing coverage.

References:

Altschul, S.F., Gish, W., Miller, W., Myers, E.W., Lipman, D.J., 1990. Basic local alignment search tool. J. Mol. Biol. 215, 403–410. https://doi.org/10.1016/S0022-2836(05)80360-2

Folmer, O., Black, M., Hoeh, W., Lutz, R., Vrijenhoek, R., 1994. DNA primers for amplification of mitochondrial cytochrome c oxidase subunit I from diverse metazoan invertebrates. Mol. Marine Biol. Biotechnol 3, 294–299.

Kearse, M., Moir, R., Wilson, A., Stones-Havas, S., Cheung, M., Sturrock, S., Buxton, S., Cooper, A., Markowitz, S., Duran, C., Thierer, T., Ashton, B., Meintjes, P., Drummond, A., 2012. Geneious Basic: an integrated and extendable desktop software platform for the organization and analysis of sequence data. Bioinformatics 28, 1647–1649. https://doi.org/10.1093/bioinformatics/bts199

Pilipenko, V., Salmela, J., Vesterinen, E., 2012. Description and DNA barcoding of *Tipula (Pterelachisus) recondita* sp. n. from the Palaearctic region (Diptera, Tipulidae). ZooKeys 192, 51–65. https://doi.org/10.3897/zookeys.192.2364

Ratnasingham, S., Hebert, P., 2007. BOLD: The Barcode of Life Data System (www.barcodinglife.org). Mol. Ecol. Notes 7, 355–364. https://doi.org/10.1111/j.1471-8286.2006.01678.x

Supplementary Article 1: Details on mock community preparation

How to prepare a 'mock community'?

Eero Vesterinen 29th June 2018

1. Pick ten individuals representing different species. Some may be closely related, but not all. Sometimes it is a good idea to use species that are not in your real samples. Sometimes it is good to use the same samples which actually represent the actual natural community.

2. Extract DNA individually from whole animals using NucleoSpin Tissue kit according to the kit manual.

3. Measure DNA concentration using Qubit.

4. Pool equimolarly; that is, the same amount of DNA from each species into the same tube per pool. Measure each pool with Qubit.

5. Use, for example, 200 ng each pool as a template in the PCR. Use mock community pools as any other sample in the metabarcoding analysis.

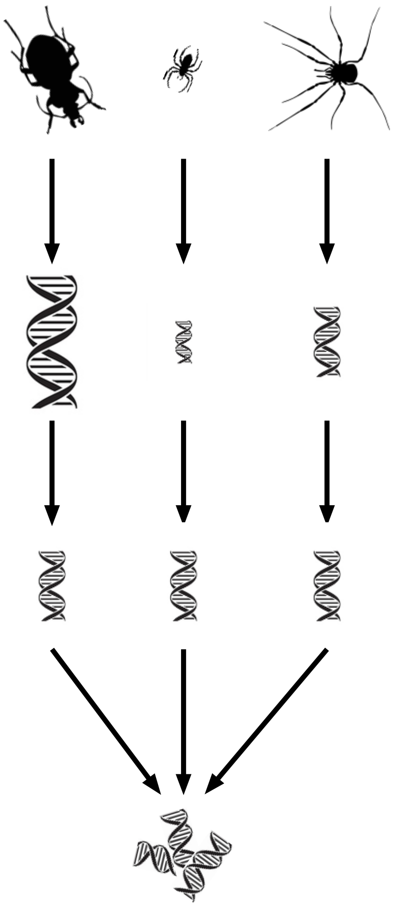

Summary figure: Summary of the mock community preparation procedure for metabarcoding analyses.
