# Supplementary material for: Metabarcoding prey DNA from fecal samples of adult dragonflies shows no predicted sex differences, and substantial inter-individual variation, in diets
Source: PeerJ. 2021 Dec 17;9:e12634. doi: 10.7717/peerj.12634 (PMC8686731; doi:10.7717/peerj.12634)
Supplement: Supplemental Information 3 — This excludes the ten most prevalent amplicon sequence variants; see main text and Table 1. Clopper–Pearson 95% confidence intervals are provided. [file peerj-09-12634-s003.pdf]

Supplementary Figure S1 for

# Metabarcoding prey DNA from fecal samples of adult dragonflies shows no predicted sex differences, and substantial inter-individual variation, in diets

**André Morrill, Kari M. Kaunisto, Julia J Mlynarek, Ella Sippola, Eero J Vesterinen, and Mark R. Forbes**

**Corresponding author: André Morrill (andre_morrill@carleton.ca)**

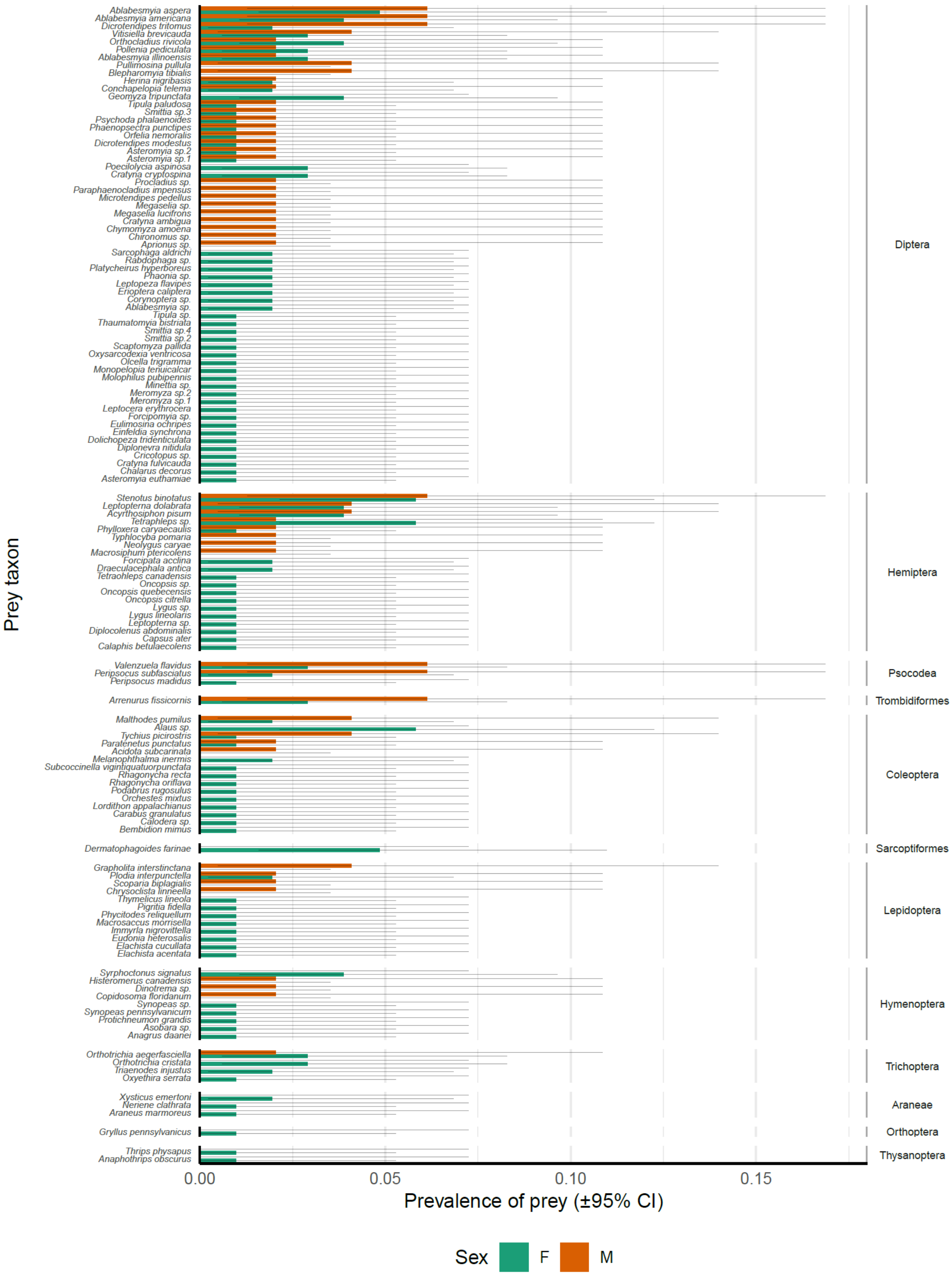
Ablabesmyia aspera
Ablabesmyia americana
Dicrotendipes tritomus
Vitisiella brevicauda
Orthocladius rivicola
Pollenia pediculata
Ablabesmyia illinoensis
Pullimosina pullula
Blepharomyia tibialis
Herina nigribasis
Conchapelopia telema
Geomyza tripunctata
Tipula paludosa
Smittia sp.3
Psychoda phalaenoides
Phaenopsectra punctipes
Orfelia nemoralis
Dicrotendipes modestus
Asteromyia sp.2
Asteromyia sp.1
Poecilolycia aspinosa
Cratyna cryptospina
Procladius sp.
Paraphaenocladius impensus
Microtendipes pedellus
Megaselia sp.
Megaselia lucifrons
Cratyna ambigua
Chymomyza amoena
Chironomus sp.
Aprionus sp.
Sarcophaga aldrichi
Rabdophaga sp.
Platycheirus hyperboreus
Phaonia sp.
Leptopeza flavipes
Erioptera caliptera
Corynoptera sp.
Ablabesmyia sp.
Tipula sp.
Thaumatomyia bistriata
Smittia sp.4
Smittia sp.2
Scaptomyza pallida
Oxysarcodexia ventricosa
Olcella trigramma
Monopelopia tenuicalcar
Molophilus pubipennis
Minettia sp.
Meromyza sp.2
Meromyza sp.1
Leptocera erythrocera
Forcipomyia sp.
Eulimosina ochripes
Einfeldia synchrona
Dolichopeza tridenticulata
Diplonevra nitidula
Cricotopus sp.
Cratyna fulvicauda
Chalarus decorus
Asteromyia euthamiae
Diptera
Stenotus binotatus
Leptopterna dolabrata
Acyrthosiphon pisum
Tetraphleps sp.
Phylloxera caryaecaulis
Typhlocyba pomaria
Neolygus caryae
Macrosiphum ptericolens
Forcipata acclina
Draeculacephala antica
Tetraohleps canadensis
Oncopsis sp.
Oncopsis quebecensis
Oncopsis citrella
Lygus sp.
Lygus lineolaris
Leptopterna sp.
Diplocolenus abdominalis
Capsus ater
Calaphis betulaecolens
Hemiptera
Valenzuela flavidus
Peripsocus subfasciatus
Peripsocus madidus
Psocodea
Arrenurus fissicornis
Trombidiformes
Malthodes pumilus
Alaus sp.
Tychius picirostris
Paratenetus punctatus
Acidota subcarinata
Melanophthalma inermis
Subcoccinella vigintiquatuorpunctata
Rhagonycha recta
Rhagonycha oriflava
Podabrus rugosulus
Orchestes mixtus
Lordithon appalachianus
Carabus granulatus
Calodera sp.
Bembidion mimus
Coleoptera
Dermatophagoides farinae
Sarcoptiformes
Grapholita interstinctana
Plodia interpunctella
Scoparia biplagialis
Chrysoclista linneella
Thymelicus lineola
Pigritia fidella
Phycitodes reliquellum
Macrosaccus morrisella
Immyrla nigrovittella
Eudonia heterosalis
Elachista cucullata
Elachista acenitata
Lepidoptera
Syrphoctonus signatus
Histeromerus canadensis
Dinotrema sp.
Copidosoma floridanum
Synopeas sp.
Synopeas pennsylvanicum
Protichneumon grandis
Asobara sp.
Anagrus daanei
Hymenoptera
Orthotrichia aegerfasciella
Orthotrichia cristata
Triaenodes injustus
Oxyethira serrata
Trichoptera
Xysticus emertoni
Neriene clathrata
Araneus marmoreus
Araneae
Gryllus pennsylvanicus
Orthoptera
Thrips physapus
Anaphothrips obscurus
Thysanoptera
Prey taxon
0.00
0.05
0.10
0.15
Prevalence of prey (±95% CI)
Sex
F
M

Figure S1: Prevalences of individual prey identified (excluding the ten most prevalent; see main text and Table 1) as amplicon sequence variants within the diets of sampled male and female *Leucorrhinia intacta*, grouped by taxonomic order. Clopper-Pearson 95% confidence intervals are provided.
